# Ligand and Residue Free Energy Perturbations Solve the Dual Binding Mode Proposal for an A_2B_AR Partial Agonist

**DOI:** 10.1021/acs.jpcb.4c07391

**Published:** 2025-01-08

**Authors:** Tana Tandarić, Hugo Gutiérrez-de-Terán

**Affiliations:** † Department of Cell and Molecular Biology, Biomedical Center, Uppsala University, Box 596, Uppsala SE-75124, Sweden; ‡ Nanomaterials and Nanotechnology Research Center (CINN), Spanish National Research Council (CSIC), Health Research Institute of Asturias (ISPA), Av. del Hospital Universitario s/n, Oviedo, Asturias ES-33011, Spain

## Abstract

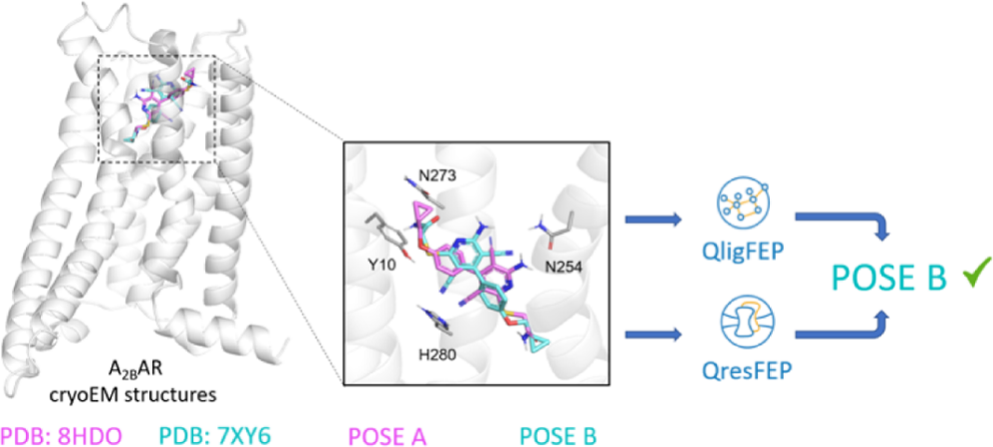

Adenosine receptors, particularly A_2B_AR, are gaining attention for their role in pathological conditions such as cancer immunotherapy, prompting the exploration for promising therapeutic applications. Despite numerous selective A_2B_AR antagonists, the lack of selective full agonists makes the partial agonist BAY60-6583 one of the most interesting activators of this receptor. Recent cryo-EM structures have univocally revealed the binding mode of nonselective ribosidic agonists such as adenosine and its derivative NECA to A_2B_AR; however, two independent structures with BAY60-6583 show alternative binding orientations, raising the question of which is the physiologically relevant binding mode. In situations such as this, computational simulations that accurately predict shifts in binding free energy can complement experimental structures. Our study combines QligFEP and QresFEP protocols to directly compare the binding affinity of BAY60-6583 between alternative binding modes as well as providing a direct comparison of in silico mutagenesis studies on each pose with experimental mutational effects. Both methods converge on the experimentally determined binding mode that better explains both the existing SAR and mutagenesis data for this ligand. Our results allow the elucidation of the experimental binding orientation that should be considered as a basis for designing partial agonist derivatives with improved affinity and selectivity and underscore the value of free energy perturbation methods in aiding structure-based drug design.

## 1 Introduction

Adenosine receptors constitute a family of G protein-coupled receptors (GPCRs), the largest group of membrane proteins found in eukaryotes, with a characteristic topology of seven transmembrane helices (7TM).^1−3^ Four members of this family, the A_1_, A_2A_, A_2B_, and A_3_ adenosine receptors (ARs) mediate responses to extracellular adenosine (ADO), the natural agonist, contributing to a variety of roles in mammalian cellular processes,^4^ including inflammation, immune response, and the sleep-wake cycle.^5−6^ Extracellular levels of ADO are usually low but can rapidly rise in response to cellular stress, offering protective effects to tissues,^7,8^ or in the case of the tumor microenvironment (TME), reducing the immune response by activation of the A_2_ group of receptors.^9,10^ Each of the four ARs displays its own distinctive features and has been extensively explored as a target for drug development, with varying levels of success.^11−13^ The A_2B_AR exhibits a markedly weaker affinity than other family members for the natural agonist ADO or derivatives, like 5′-*N*-ethylcarboxamidoadenosine (NECA).^14^ The pathophysiological relevance of A_2B_AR has been only recently recognized due to its overexpression under pathological conditions,^15^ where extracellular ADO levels rise dramatically, enabling A_2B_AR activation.^7,8,16^ Consequently, the therapeutic potential of the A_2B_AR sparked in conditions such as inflammation,^17,18^ diabetes,^19^ pain,^20^ asthma,^21,22^ Alzheimer’s disease,^23,24^ and cancer,^25,26^ with a succulent chemical repertoire of selective A_2B_AR antagonists.^27−33^ Within the cancer area, we recently demonstrated the promising potential of A_2B_AR antagonism in revitalizing the immune system in the TME,^34^ and several A_2B_AR antagonists are currently undergoing clinical trials.^35−37^

**Figure 1:**
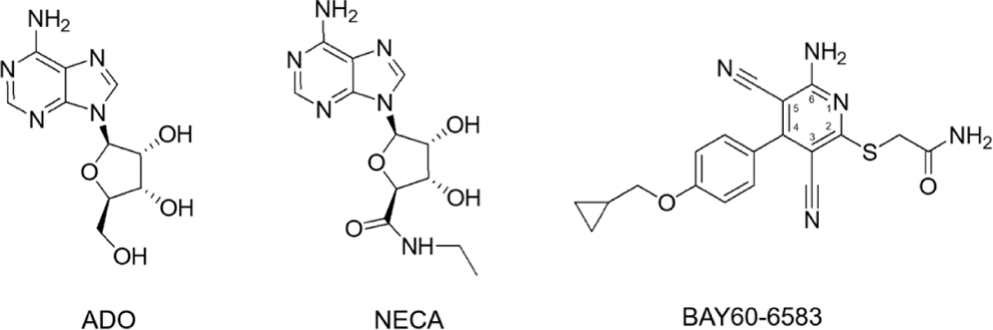
Chemical structures of the A_2B_AR agonists (ADO, NECA) and partial agonist (BAY60-6583 60-6583) discussed in this work.

Atomic resolution X-ray crystallography and Cryo-EM structures have significantly contributed to our increased comprehension of the functionality of the different ARs, allowing the structure-based design of compounds targeting either orthosteric or allosteric binding sites to modulate adenosine receptor function.^38^ The structures of both A_1_AR and A_2A_AR receptor have been resolved in active, inactive, and intermediate conformations with a wide range of orthosteric ligands.^39−40^ In contrast, the structures of A_2B_AR^41,44^ and A_3_AR^45^ have only been recently resolved by cryo-EM in their active conformation. In the particular case of the A_2B_AR, these include complexes with both full agonists ADO and NECA, as well as with the nonribosidic, partial agonist BAY60-6583 (Figure 1). This molecule and other analogues of the 2-aminopyridine-3,5-dicarbonitrile scaffold had been initially postulated as full agonists^46^ but were later characterized as selective, partial agonists for the A_2B_AR.^47^ BAY60-6583 is indeed one of the few selective A_2B_AR agonists identified so far,^48^ demonstrating a protective effect in treatment following ischemic injury^49^ and skin inflammatory diseases such as psoriasis.^50^ Beyond this therapeutic interest, there is an increased need for potent and selective agonists to better characterize the pathophysiological role of the A_2B_AR.^37^

**Figure 2:**
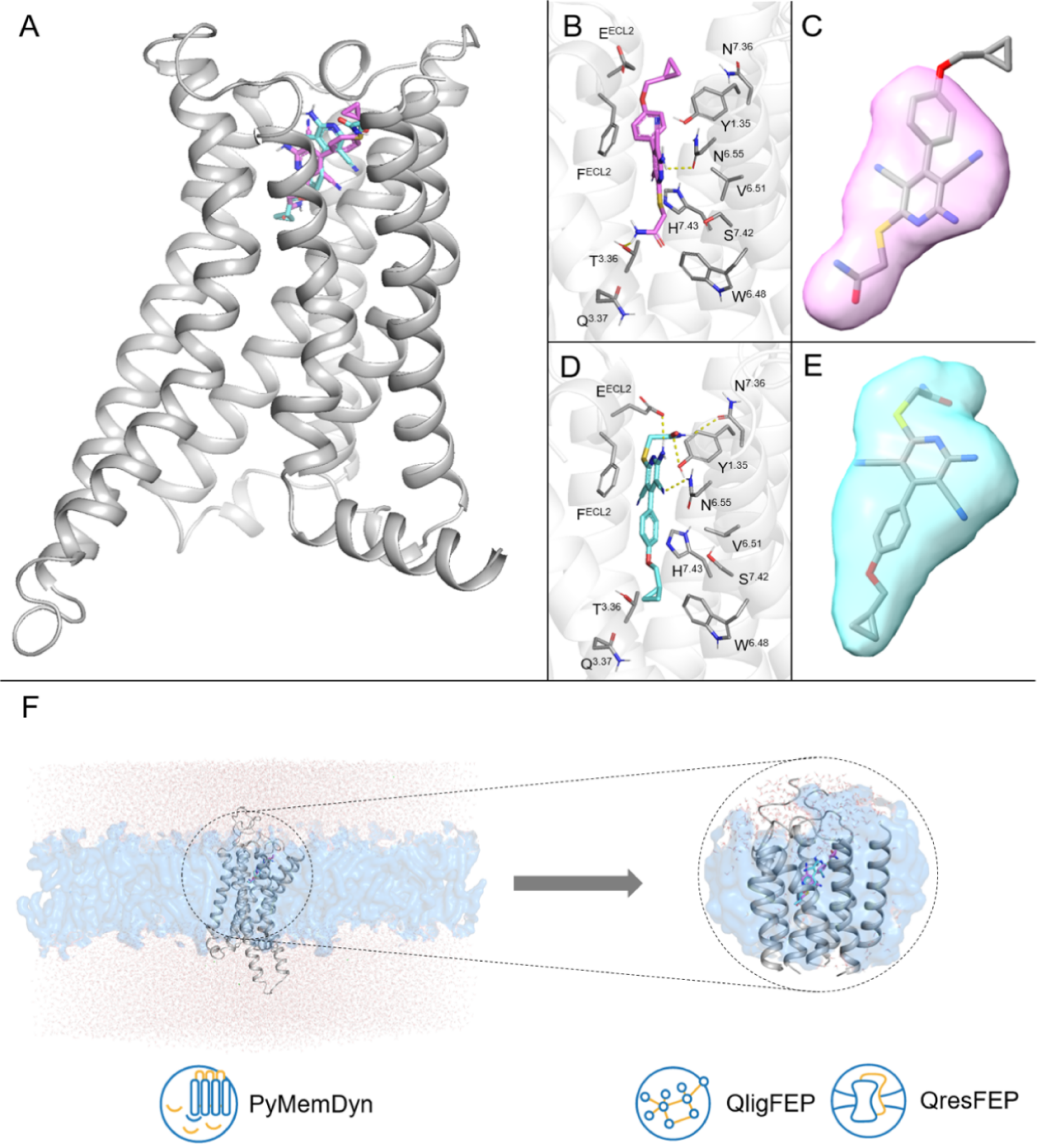
(A) Superposition of the experimentally proposed binding modes of the partial agonist BAY60-6583 to the active conformation of the A_2B_AR. (B) Inset of the herein referred to as pose A, corresponding to the structure with PDB code 8HDO(41) (magenta, 2.87 Å resolution) and (C) the corresponding cryo-EM density map. (D) Pose B (cyan, 2.99 Å resolution) is extracted from cryo-EM structure with PDB code 7XY6(44) alongside with (E) the corresponding Cryo-EM density map. Hydrogen bonds described in the corresponding structures are shown in yellow. (F) Pipeline followed in this work for the MD simulations, under periodic boundary conditions, and subsequent FEP calculations, under spherical boundary conditions (see Section 2).

While previous homology-based models allowed us and others to explore the structural dynamics of the A_2B_AR^52−54^ and highlight the mutations responsible for receptor specificity for individual ligands,^51−53^ the new experimental structures open the door to more accurate structure-based, computer-aided ligand design. However, Cryo-EM structures of GPCRs, falling within the 2.2–4.6 Å resolution range, show their highest resolution in the interface between protein–protein interactions and not usually within the ligand binding site, occasionally resulting in ambiguous or incomplete model building of the ligand binding region. Tools for automatically fitting small molecule ligands into Cryo-EM data often rely on the shape and topological features of density maps, leading to imprecise binding mode determinations, especially in the case of somewhat symmetrical molecules.^54^ In this sense, the recent complexes of A_2B_AR with ADO and NECA compare well with the extensively characterized binding mode of these and other ribose-containing full agonists on the remaining ARs.^39,42,45^ In contrast, the structures with the selective partial agonist BAY60-6583^41,44^ show alternative binding modes with a flip of the ligand along the long axis of its molecular structure (Figure 2). This raises the question of whether these represent two potential binding modes or indicate a structure that is ambiguously resolved.

Computational methods to accurately estimate shifts in binding free energy provide the missing link between structural changes in ligands or point mutations in the receptor and the experimental binding affinities determined for protein–ligand complexes. For years, rigorous free energy perturbation (FEP) methods have been employed to elucidate structure–affinity relationships (SARs) surrounding a specific chemical framework.^55,56^ Recent advances have made it feasible to routinely apply this approach in ligand design,^57^ with protocols that can also be leveraged to evaluate changes in binding free energy resulting from site-directed mutagenesis (SDM) data.^58^ By combining ligand and residue FEP simulations, a comprehensive energetic map of the molecular interactions dictating protein–ligand binding can be obtained. This forms the basis of two complementary protocols developed in our laboratory, namely QligFEP^59^ and QresFEP,^60^ integrated into the molecular dynamics (MD) software package Q.^61^ The first integrated approach of ligand and residue FEP simulations was published by Boukharta et al. to characterize antagonist binding to the Y1 neuropeptide receptor,^62^ which we later expanded to other GPCRs, including the family of adenosine receptors.^63^ Our integrated residue and ligand FEP approach guided further chemical synthesis and biophysical mapping to reveal the binding mode of a novel antagonist series to the A_2A_AR confirmed by X-ray crystallography.^64^

In this work, we analyze the two experimentally proposed binding modes of BAY60-6583 to the A_2B_AR to reconcile discrepancies and propose and characterize the most probable binding mode of this ligand that explains the existing site-directed mutagenesis (SDM) and structure–activity relationship (SAR) data reported for this scaffold. To do so, we followed a protocol consisting of extensive MD simulations on agonist-bound A_2B_AR structures, followed by estimation of the binding affinity difference between the binding orientations of BAY60-6583, and finally an integrated FEP approach to explore the SAR and SDM on both the wild-type (WT) A_2B_AR as well as simulate a number of receptor mutants (Figure 2F).

## 2 Methods

### 2.1 System Preparation

The following cryo-EM structures of human A_2B_AR were retrieved from the Protein Data Bank, in all cases representing the active conformation of the ternary complex with an engineered G_s_ protein and different agonist molecules: the partial agonist BAY60-6583 and (PDB code 8HDO(41) and 7XY6(44)) and the full agonist NECA (PDB code 7XY7(44)). All structures were prepared as follows: (i) the engineered G_s_ protein was removed. (ii) The missing loops were rebuilt using Modeler,^65^ while the disulfide bridges were modeled following the definition in the corresponding structural article.^41,44^ (iii) Protonation states of titratable side chains were assigned using the Schrödinger Maestro Protein preparation tool,^66^ aiming to optimize the internal hydrogen bond network. In the BAY60-6583 systems, both charged (HIP) and δ-protonated histidine (HID) models were considered for His280^7^·^43^. (iv) The water network within the ribose binding site was adopted from the crystal structure of agonist-bound A_2A_AR (PDB code: 2YDV(67)), following a superposition of both structures with Pymol^68^ and retaining the water molecules interacting with conserved residues (six in the case of A_2B_AR-BAY60-6583 complexes, seven in the case of A_2B_AR-NECA).

### 2.2 MD Simulations

All protein ligand systems, together with corresponding apo structures (generated by removing the ligand), were subsequently equilibrated in a solvated membrane using the PyMemDyn automated protocol.^69^ In short, the receptor structure is embedded with the TM bundle parallel to the vertical axis of a pre-equilibrated POPC (1-palmitoyl-2-oleoylphosphatidylcholine) membrane model, soaked with bulk SPC^70^ water and centered on a hexagonal prism-shaped box, which is then energy minimized prior to a 5 ns molecular dynamics (MD) equilibration with GROMACS.^71^ MD equilibration occurs in the framework of periodic boundary conditions (PBC) in the NPT ensemble, using the OPLSAA force field for the protein there implemented^72^ and ligand (calculated with the LigParGen software^73^) in combination with the Berger parameters^74^ for the POPC lipids. Positional restraints on all heavy atoms of the protein are gradually released from 1000 to 200 kJ·mol^–1^·nm^–2^ (in five stages of 0.5 ns each) in an initial phase of “solvent and membrane equilibration” followed by 2.5 ns where the force constant of 200 kJ·mol^–1^·nm^–2^ was applied on the Cα trace, while a force constant of 1000 kJ·mol^–1^·nm^–2^ is retained on the ligand heavy atoms during the whole equilibration phase. The production phase extended for triplicate unrestrained MD simulations of 100 ns length each using GROMACS,^71^ along with the utilization of the half-ε double-pairlist method,^75^ Nose–Hoover thermostat^79^ targeting a physiological temperature of 310 K,^76^ 2 fs time step alongside the LINCS algorithm to constrain the bonds involving hydrogens.^77^ Electrostatic interactions beyond a 12 Å cutoff were approximated with the particle mesh Ewald method.^78^ Subsequent MD analyses were conducted utilizing various GROMACS and VMD^79^ utilities as well as the MDtraj,^80^ MDAnalysis,^81^ and mdciao^82^ Python modules, while molecular superimpositions, trajectory visualizations, and molecular images were generated using PyMOL.^68^

### 2.3 FEP Simulations

The system after the “solvent and membrane equilibration” phase is extracted as a PDB file and transferred to the Q software for free energy simulations under spherical boundary conditions,^61,83^ using the surface-constrained all-atom solvent (SCAAS) model,^84^ as illustrated in Figure 2F. The main advantage of such an aperiodic finite system for free energy calculations is its superior computational efficiency, with a typical size of the sphere being 5,000 atoms, as opposed to ∼50,000 atoms of a GPCR under PBC. In addition, it avoids sampling of highly unstructured and irrelevant regions, such as intracellular loops, contributing to increased convergence in simulation times that are typically of a few ns, allowing us to sample 10 individual replicate simulations and estimate associated errors. Two automated protocols were applied to set up, run, and analyze free energy perturbation (FEP) simulations: QligFEP^59^ for ligand perturbations and QresFEP^60^ to model SDM effects on ligand binding. Both FEP protocols are freely available at https://github.com/qusers/qligfep.

In all cases, a 50 Å diameter simulation sphere was defined, centered on the geometric center of the ligand for QligFEP, or the residue undergoing mutation for the QresFEP protocol. Radial and polarization restraints were imposed on solvent atoms following the SCAAS model to replicate bulk water properties at the sphere’s surface.^61,84^ Boundary solute atoms within the outer 3.5 Å layer of the sphere were slightly constrained, with a 10 kcal·mol^–1^·Å^–2^ force constant, while atoms outside the sphere were tightly constrained with a 200 kcal·mol^–1^·Å^–2^ force constant and were disregarded in the calculation of nonbonded interactions. Long-range interactions beyond a 10 Å cutoff and within the simulation sphere were approximated using the local reaction field method,^85^ except for atoms undergoing FEP transformation, where no cutoff was applied. Solvent bonds and angles were constrained using the SHAKE algorithm.^86^ Ionizable residues outside the sphere and those within the boundary were considered in their neutral form, while those inside the sphere were modeled in their default, charged form.^59^ Histidine residues were modeled in their δ protonation state except His280^7^·^43^ where both δ and positively charged versions were considered in parallel simulation setups.^55^ The OPLSAA/M force field, with improved protein torsional parameters,^87^ was adopted for the receptor together with compatible ligand parameters obtained from Schrödinger’s ffld_server^88^ and translated to Q following the QligFEP protocol. The simulation sphere underwent a restrained equilibration period, where temperature increased from 0.1 to 298 K, the target room temperature mimicking conditions of the EC_50_ assays, while positional restraints on heavy atoms were gradually released from 25 kcal·mol^–1^·Å^–2^. It follows an unrestrained and unbiased equilibration preceding FEP sampling, wherein atom transformations occurred between the initial and ending states, divided into λ windows of 10 ps each. The number of lambda windows, their distribution (evenly or sigmoidal), and total sampling differed from QligFEP (ligand perturbation) to QresFEP (residue perturbation) simulation, as depicted below. In all cases, the total sampling consisted of 10 independent MD simulations with varying initial velocities, with average values reported together with associated standard errors of the mean (, where σ = standard deviation; *n* = 10; with the total error, ).

Ligand perturbations with QligFEP compared alternate binding modes of BAY60-6583, or transformations of this ligand to a series of analogues, in either binding mode. The starting point was the equilibrated BAY60-6583-A_2B_AR complex corresponding to one pose, while the second pose was generated by structural superposition of the alternative binding mode as deposited in the PDB with PyMol,^68^ retaining only the ligand (for binding mode comparison). For the ligand series FEP studies, the preparation of the second ligand in analogous conformation was performed with Schrödinger’s Maestro software, in all cases followed by treatment with LigPrep.^89^ QligFEP^61^ dual topology approach is then run with the following parameters: 100 λ windows with sigmoidal distribution, ensuring a denser sampling toward the endpoints, and starting in the middle (λ = 0.5). A set of pairs of topologically equivalent atoms was manually defined, based on the superposition of alternate poses (see Figure S1), and half-harmonic distance restraints (5.0 kcal/mol/Å^2^) were applied to keep each pair within a window distance of 0.0–0.2 Å. For the FEP transformation along ligand series, we used the automated restraints defined by QligFEP. One particularity of the FEP application to rank binding modes (L1 and L1′) is that the water leg of the thermodynamic cycle can be obeyed, since such transformation should give a theoretical value of Δ*G*_w(L1→L1′)_ = 0; thus, the relative free energy is directly estimated from the Δ*G*_P(L1→L1′)_ using the BAR approach.^90^ Total simulation time (excluding equilibration) per ligand FEP transformation was 2 (water/protein) × 10 replicas × 100 λ × 10 ps = 20 ns (10 ns for ranking binding poses, lacking the water leg).

The single-topology FEP protocol for amino acid perturbations, QresFEP, breaks down the side chain perturbation to alanine into separate stages. During these stages, atom annihilations occur gradually for each charge group, as defined by the OPLS force field, beginning with the atom most topologically distant from C_β_.^58,62^ The Initial stage of FEP simulations involves neutralization of all partial charges of the most distal charge group, followed by annihilation of the vdW potentials of that charge atom, while the same process (i.e., neutralization of partial charges) starts on the next charge group and so on until the from C_β_. The number of FEP stages needed to annihilate the whole side chain varies depending on the size and number of charge groups involved, ranging from four (Ser) to nine (Trp) FEP stages, each of them consisting of 50 λ windows, yielding simulation times per Ala mutation of: 2 (holo/apo) × 10 replicas × 4–9 stages × 50 λ × 10 ps = 40–90 ns. To complete the thermodynamic cycle, the same side chain annihilation is also simulated in the apo structure of the protein so that the energy difference between these two processes equals the binding affinity shift due to mutation. Non-Ala mutations involve fulfilling two thermodynamic cycles in opposite directions, which are additive (WT → Ala ← Mut). Relative binding free energies (ΔΔ*G*) were estimated by solving the thermodynamic cycle using the Bennett acceptance ratio method (BAR).^90^

### 2.4 Molecular Docking

Redocking of BAY60-6583 was performed in both A_2B_AR experimental structures reported with this ligand,^92^ considering both δ-protonated and positively charged His280^6^·^43^ in four parallel docking simulations. Receptor structures were prepared as described in Section 2.1, and automated docking was performed with the Schrödinger Glide module.^91^ The docking grid was defined by taking as a reference the experimental coordinates of BAY60-6583 in each case, expanding the cubic grid box 15 Å in each dimension to cover the whole orthosteric pocket. A maximum of 50 output docking poses were requested in each of the four runs, and for each of them, the heavy-atom RMSD was calculated relative to a reference position, in our case, the experimental pose within PDB code 7XY6. The RMSD matrix was used as a basis for *k*-means clustering of all resulting poses.

## 3 Results and Discussion

Initial comparison of the two cryo-EM structures of A_2B_AR with partial agonist BAY60-6583 revealed that the two experimentally proposed binding modes (i.e., pose A, PDB code 8HDO, Figure 2B in magenta, and pose B, PDB code 7XY6, Figure 2D in cyan) are related by a flip of the molecule along the long axis. Consequently, the two rings of the ligand core swap positions, leading to an interchange of the binding sites occupied by the cyclopropyl and the amide substituents, together with rearrangement of the positions occupied by the exocyclic amino and the neighboring cyano groups, mostly interacting with Asn254^6^·^55^ on the two poses (Figure 2). Comparison of the electron density maps, however, reveals that pose A (PDB 8HDO, Figure 2C) has no real density for the solvent-exposed cyclopropyl substituent. A related difference is located in the extracellular loop region of the receptor, in particular the conformation of the side chain of Glu174^EL2^, which only in pose B (PDB code 7XY6, Figure 2E) is making contacts with the exocyclic amino group of the ligand, while in pose A (PDB code 8HDO, Figure 2C), this side chain is modeled on an alternative conformer, looking toward the extracellular side to avoid electrostatic repulsion with the partial negative charge of the cyano group of the ligand. Finally, the interactions of the remaining cyano group with the neighboring His280^7^·^43^ are somehow conserved between the two poses, but a detailed analysis would advise for a double-protonated (charged) histidine in pose A (Figure 2B), while for pose B (Figure 2D), the interaction might be mediated by a water molecule as the distance seems too long for a direct H-bond (4.6 Å). Thus, alternative protonation states of this residue were considered, following previous proposals on related A_2A_AR.^53^

The first computational investigation to assess these alternative binding modes consisted of redocking the ligand BAY60-6583 in both A_2B_AR structures reported in complex with this molecule. The pool of docking poses could be clustered into three groups, based on the RMSD with respect to a reference structure. The least populated cluster corresponds to pose A, with a second, much more populated cluster aligning with pose B, and finally a third cluster (from here on pose C, the most populated) is somehow analogous to pose B with the cyclopropyl ring positioned inside the receptor, differing in a relative flip of the pyridine ring (see Figure S2). All three clusters preserved otherwise the two key interactions corresponding to a hydrogen bond with Asn245^6^·^55^ and π–π stacking with Phe173^ECL2^ and were not really distinguishable attending to the best docking score per pose, irrespective of the receptor structure (i.e., PDB code 7XY6 or 8HDO) or protonation state considered for His280^7^·^43^ (HID or HIP), with the highest overall docking score achieved for pose B as obtained with the 8HDO/Hip280^7.43^ system (see Figure S2). Interestingly, docking to 7XY6 did not generate any pose A orientation, most probably due to the particular conformer adopted by Glu174^ECL2^, while poses B and C occurred with comparable frequency, with pose B having a slightly higher docking score (see Table S1). While providing a first insight into the preference of pose B, docking results are inconclusive, in line with previous docking reports of BAY60-6583 and derivatives: the Müller lab initially proposed indeed pose A,^51^ based on docking performed on homology models of inactive A_2B_AR and later reproduced on partially active regenerated models of the A_2B_AR.^92^ On the other hand, the SAR of a novel series of aminopyridine-3,5-dicarbonitrile derivatives reported by the Colotta group could be rationalized using a binding mode analogous to pose B of BAY60-6583.^46^

We then proceeded to analyze the stability of the structures and receptor–ligand interactions via classical MD simulations. We also included here the complex of A_2B_AR with NECA (PDB code 7XY7) as a control, since the ligand adopts the well-known binding mode of adenosine-derived agonists in the remaining adenosine receptors.^43,67,94^ The binding orientation of NECA was quite stable, with an average ligand RMSD of 0.83 Å (see Figure S3), Hence, the ribose ring maintains direct or water-bridged hydrogen bonds between O3′ and Ser279^7.42^ and O2′ and His280^7.43^, with the carboxamide group maintaining a tight hydrogen bond with Thr89^3.36^ (see Figure S4). Additionally, NECA makes hydrophobic contacts with Leu86^3.33^, Trp247^6.48^, Met182^5.38^, Ile276^7.39^ along with the π–π stacking of the purine ring with Phe193^ECL2^. These interactions are highly conserved within the adenosine receptor family, as we previously reported on simulations of NECA with the A_2A_AR,^95^ with the primary difference between the A_2B_AR orthosteric site and other adenosine receptors being Val250^6.51^, a position occupied by a bulkier leucine in the other adenosine receptors. As compared to the experimental structure of A_2A_AR in complex with NECA^94^ (PDB code 6GDG), one can appreciate changes in the rotameric state of His251^6.52^, which no longer makes a hydrogen bond with the carboxamide group of NECA in the cryo-EM structure of A_2B_AR. This is reflected in the low frequency of this interaction during MD simulations. The conserved hydrogen bond between the exocyclic amino group and Asn254^6.55^ is also less frequent and instead replaced by a water-mediated H-bond interaction. Simultaneously, we observed a relatively high frequency of the second H-bond between the exocyclic amine group with the conserved Glu174^ECL2^ (a side chain that was originally not modeled in the cryo-EM structure).

**Figure 3:**
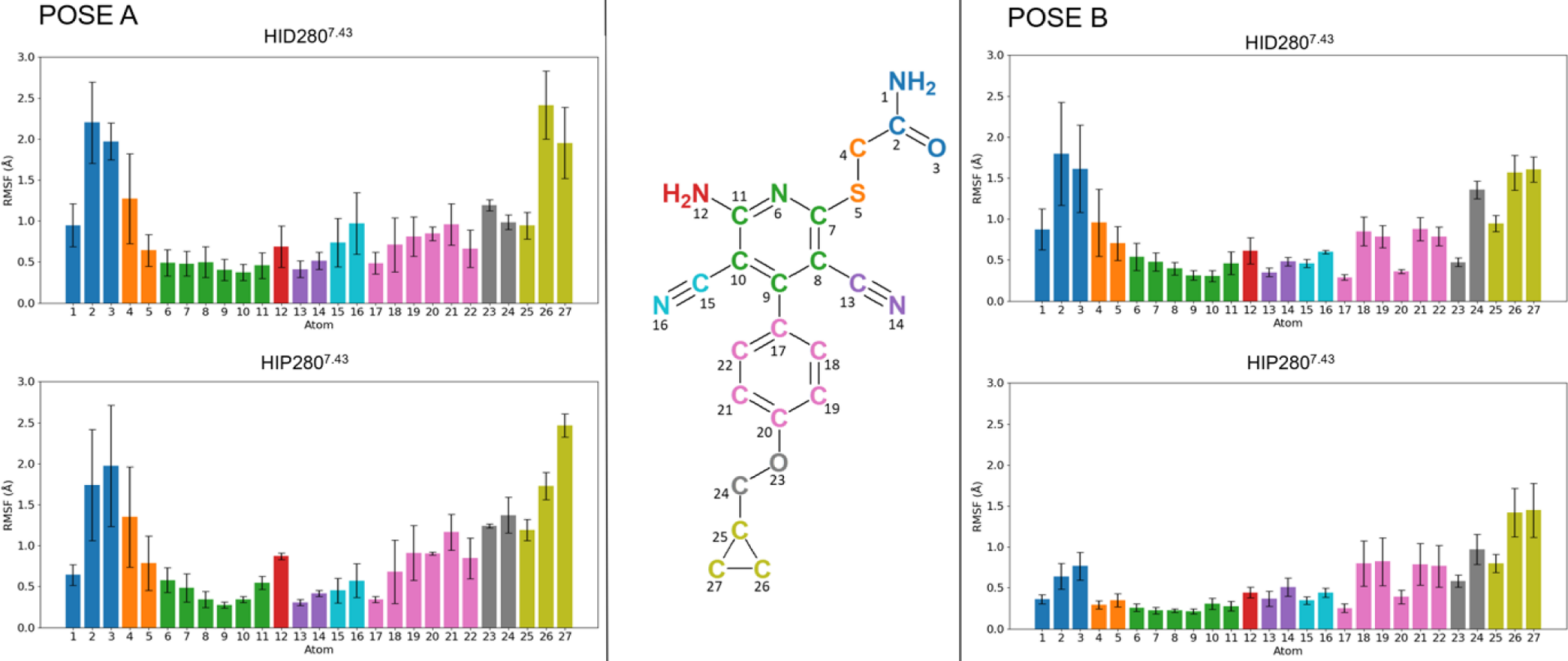
RMSF analysis of BAY60-6583 under different MD simulation conditions. Bar plots are colored following the color scheme and atom numbering of the 2D representation of the molecule (in the center) to facilitate the identification of the different chemical groups.

For each of the two structures of the A_2B_AR in complex with BAY60-6583, we performed parallel MD simulations considering alternate protonation states of His280^7.43^ (HID or HIP), which together with the NECA simulation above yields a total of 1.5 μs MD sampling of the A_2B_AR complexes. The fluctuations of BAY60-6583 along the two sets of triplicate MD simulations are in all four cases quite low (RMSD below 2 Å, Figure S5). A more informative ligand RMSF analysis with the combination pose B_HIP shows remarkably higher ligand stability (Figure 3). As expected, in all cases, the flexibility is higher for the 6-sulfanylamide (blue and orange bars, Figure 3) and the para-cyclopropylmethyloxyphenyl (light green, gray, magenta, Figure 3) groups, while the core of the ligand is anchored by polar interactions. However, one can immediately appreciate that both substituents find a better accommodation on pose B_HIP as compared to other binding models.

**Figure 4:**
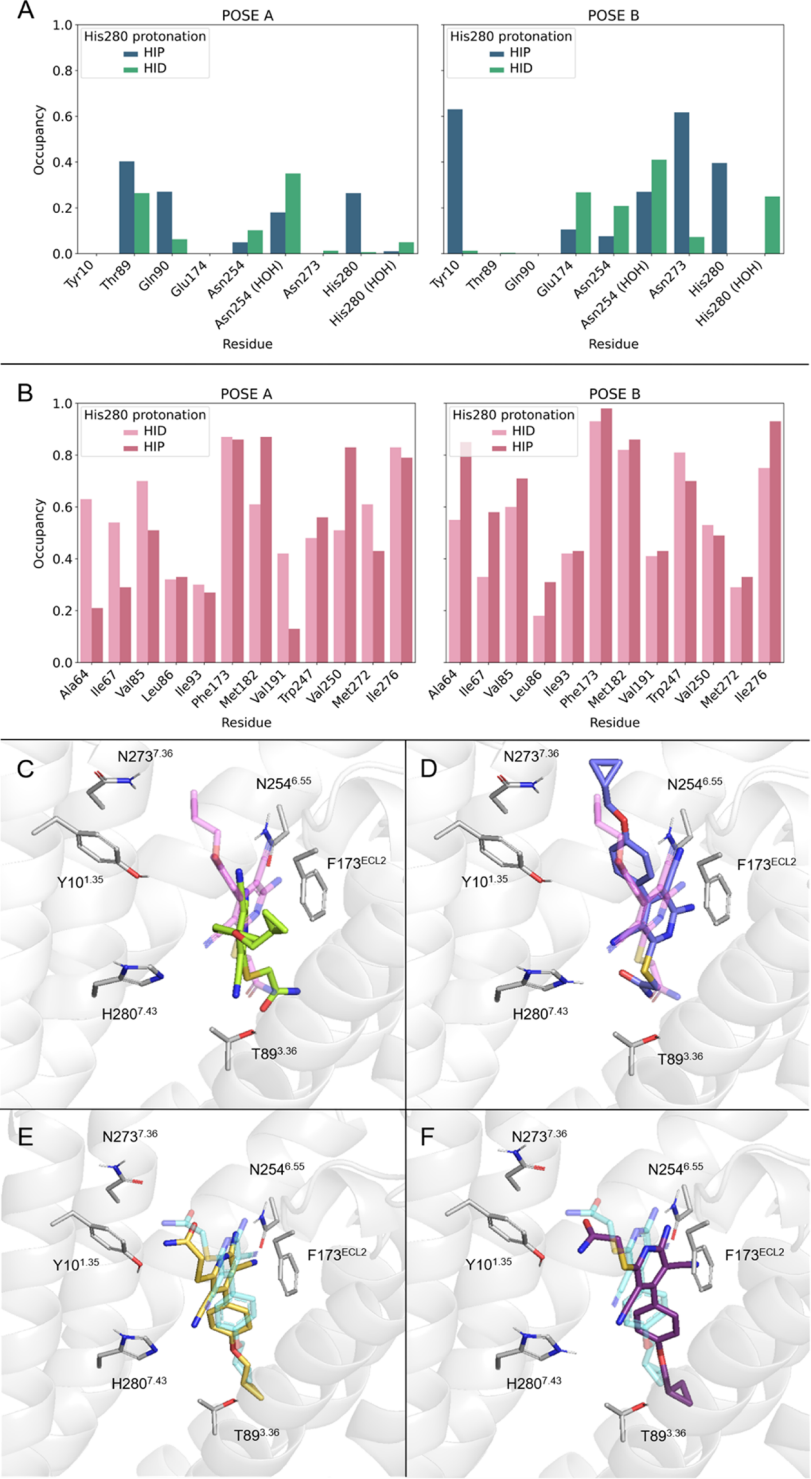
Contact analysis as extracted from MD simulations. (A) H-bond occupancy by residue (pose A, left; pose B, right), considering both protonation states of His280^7.43^ (neutral δ-protonated, HID, green; positively charged, HIP, blue). (B) Hydrophobic contact occupancy by residue, defined as hydrophobic side chains that have any atom within distance *d* < 3.8 Å of any ligand atom (HID, light salmon, HIP, dark salmon). (C–F) Representative structures from the MD simulations superimposed with the corresponding experimental structure (C) pose A, HID, (D) pose A, HIP, (E) pose B, HID, (F) pose B, HIP.

To gain more insight into the specific contacts governing each binding mode, we performed a comparative analysis of hydrogen bond (H-bond) and hydrophobic interactions of all four MD simulations. For the purposes of this analysis, the three independent MD replica simulations were joined into a linear 300 ns sampling time. The results are depicted in Figure 4, together with representative structures from MD simulations selected as the center of the most populated of the *k*-means cluster performed over a PCA. Pose B_HIP maintains a direct H-bond between Hε of His280^7.43^ and the cyano group at position 3, reinforcing the polar interactions of the methylamide with Asn273^7.36^ and Tyr10^1.35^, as highlighted by Chen et al. using pose B (Figure 4A).^44^ Analogous MD simulations of pose B with the HID setup showed a water-mediated interaction between the cyano group and His280^7.43^, and a much lower frequency of the interactions with the methylamino group. In both cases, the core of the ligand is anchored either through direct or water-mediated H-bond with Asn254^6.55^ and π–π stacking of the pyridine ring with Phe173^ECL2^, as it is usually the case on AR ligands, while the cyclopropyl substituent is binding within the hydrophobic cavity defined by Trp247^6.48^, the “toggle switch” involved in receptor activation, Val191^5.47^ and Ile93^3.40^ (Figure 4B). Conversely, the MD simulations of pose A do not show significant differences between the HID and HIP setup, beyond the direct interactions between Hε of His280^7.43^ and the other cyano group in position 5 of the ligand, which otherwise is too close to the partial negative charge of the Nε in HID, not allowing a water-mediated interaction either. In both cases, the methylamino group slightly sinks in pose A toward the inner cavity of the receptor, to present relatively frequent H-bonds with Thr89^3.36^ and Gln90^3.37^ (Figure 4A). The direct or water-mediated hydrogen bond of the exocyclic amino group with Asn254^6.55^ is slightly less frequent in pose A, following the unfavored geometry for this interaction in the experimental structure. Taking the HIP setup as a reference, the average occupancy of hydrophobic interactions is higher in pose B (0.63 ± 0.04, measured on the 3 replica simulations independently) than in pose A (0.55 ± 0.04), with a statistical significance of *p* < 0.05, reinforcing the idea that the accommodation of the cyclopropyl substituent is optimal in the inner binding pocket defined in pose B.

To further assess the physiologically relevant binding orientation, we quantified relative binding free energies (RBFEs) under different conditions with an integrated ligand and residue free energy perturbation (FEP) protocol. A first exploration consisted of a direct comparison of the ligand binding free energy difference between the two orientations. As opposed to most ligand FEP applications, restricted to exploring substituent changes on a common scaffold, our representation of the ligands in separate topologies allows a direct ranking of binding modes based on estimated differences in their free energy of binding. A previous application of this approach on the related A_2A_AR allowed us to correctly reproduce the experimental data reporting the dual binding mode of caffeine, while the single methylation leading to theophylline shifted the preference toward one orientation, again in agreement with experimental data.^64^ This proof of concept preceded the application of QligFEP on a prospective study on the elucidation of the binding mode of a pharmaceutical series of chromone derivatives on this receptor, the results of which were validated with the corresponding crystal structures.^64^ Analogous implementations of this approach on our A_2B_AR inactive homology model allowed us to univocally identify the favored enantiomer in a series of enantiospecific antagonists from our design program.^96,97^ The direct FEP comparison between pose A and pose B of BAY60-6583 was examined under the same four conditions that we considered in the classical MD simulations, the results being summarized in Table 2. In all cases, the binding mode in pose B is strongly favored, with predicted affinity differences in the order of 8.8–10.9 kcal·mol^–1^, and reasonable SEM values in the order of 0.7–0.8 kcal·mol^–1^ in line with our previous results of direct pose comparison (Table 1).^64^

**Table 1:** Calculated Free Energy Difference (ΔΔ*G*_calc_ [kcal·mol^–1^]) between Pose A and Pose B of BAY60-6583

	Pose comparison B → A (ΔΔ*G*_A–B_, kcal·mol^–1^)	
	8HDO	7XY6
HID280^7.43^	10.97 ± 0.76	8.81 ± 0.79
HIP280^7.43^	9.93 ± 0.71	10.66 ± 0.81

During all four FEP simulations, compound BAY60-6583 in pose B forms H-bonds alternatively with Asn273^7.36^ and Tyr10^1.35^, which is consistent with mutational studies highlighting the significance of these two amino acids in BAY60-6583 binding.^44^ The cyclopropyl group makes favorable vdW interactions with the residues conforming the hydrophobic pocket formed by Trp247^6.48^, Val191^5.47^, and Ile93^3.40^, in agreement with the observations from the plain MD simulations reported above. In both simulations considering double-protonated His280^7.43^, the ε-NH group of this histidine forms an H-bond with the cyano group in either position 3 (pose B) or position 5 (pose A) of the ligand. Generally speaking, these and other polar interactions follow the same pattern as described in the unbiased MD simulations section. The only noticeable difference is the disturbance of the π–π stacking of the aminopyridine-3,5-dicarbonitrile core with Phe173^ECL2^ in pose A, due to the bending of the unfavored, solvent-exposed hydrophobic cyclopropyl moiety toward the contiguous phenyl ring and leaning toward Ile276^7.39^.

The binding mode determined for BAY60–6583 to A_2B_AR should explain the existing structure–activity relationship (SAR) established for congeneric series. To probe this aspect, we recovered the series of A_2B_AR partial agonists reported by the Colotta group,^46^ developed to evaluate the effects on ligand potency of chemical modifications on the 6-sulfanyl substituent of the aminopyridine-3,5-dicarbonitrile chemotype, or replacements of the para-cyclo-propylmethyloxy group on the 4-aryl substituent. From the derivatives with experimental EC_50_ values in that study, only single substitutions on either of these two positions were considered for QligFEP calculations, consisting of pairwise RBFE comparisons with the parent BAY60-6583, as shown in Figure 4. The same star-like QligFEP set of simulations was run in parallel on both HID or HIP setups, in each case for pose A and pose B. The results are in line with the direct FEP transformations of BAY60-6583, showing a very nice agreement with experimental potency shifts when simulations are run on pose B_HIP (mean unassigned error, MUE = 1.15 kcal·mol^–1^, *r*^2^ = 0.59, Figure 4) as compared to the corresponding simulations on pose A_HIP (MUE = 1.61 kcal·mol^–1^, *r*^2^ = 0.004, Figure S9). The corresponding results using a neutral histidine setup (HID) showed in both cases less predictive power (see Figures S10 and S11). The ligand FEP simulations here reported were performed over a total of 0.48 μs of simulation time (see Section 2 for details on how to calculate this number).

**Figure 5:**
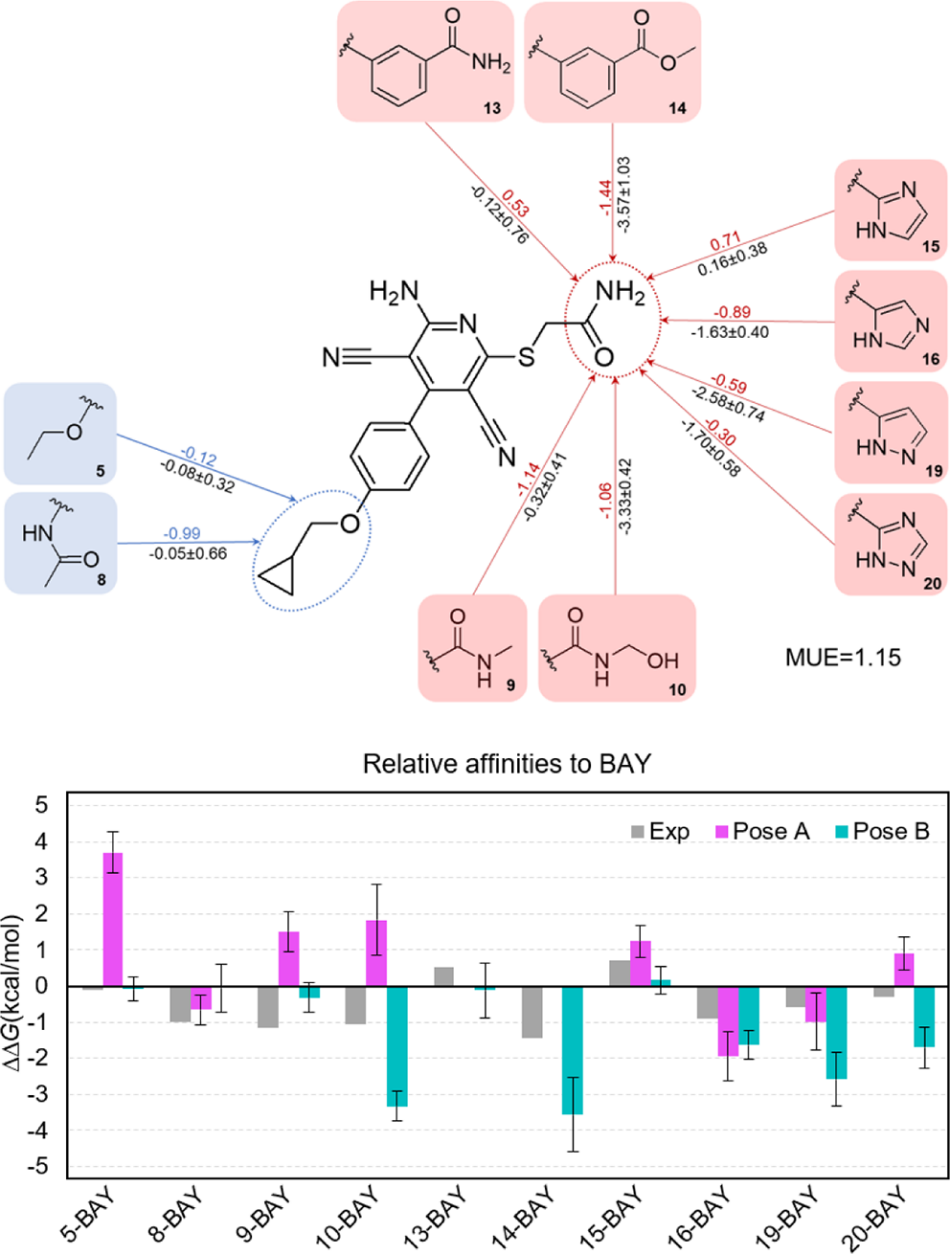
RBFE calculations of a series of one-site analogues extracted from ref (46) (see text), as compared to BAY60-6583. (Top) Simulations were run on pose B, in the presence of Hip280^7.41^. The calculated numbers (ΔΔ*G*_L-BAY_, kcal·mol^–1^) follow the direction indicated by the arrow, with FEP values in black (with associated SEM), and experimental values derived from EC_50_ in colored font. (Bottom) Comparative analysis of the values obtained for pose B_HIP (cyan, values from top panel), pose A_HIP (magenta, values in Figure S9) together with the experimental values (gray bars).

Substitutions of the 6-sulfanyl group introduce in all cases hydrogen bond donor and acceptor groups (Figure 5), either by bioisosteric replacement of the carboxamide (**cmpds 9–14**), or by the incorporation of heterocycles such as imidazole (**cmpds 15–19**) and triazole (**cmpd 20**). When simulated on pose B, we observed that the introduced groups generally tend to form hydrogen bonds with Tyr10^1.35^ and/or Asn273^7.36^, while maintaining the scaffold interactions previously described for BAY60-6583. The predominant effect of these substitutions is to moderately reduce the affinity compared to BAY60-6583, which is correctly predicted in our simulations. The series contains, however, two cases of experimental affinity gain: **cmpd 14**, bearing the aryl homologation of the carboxamido function, has a neutral effect in our calculations, while the corresponding ester analogue in **cmpd 13** effectively reduces the affinity in both experimental and calculated data. The highest gain in affinity corresponds to the imidazole substitution on **cmpd 15**, which is correctly predicted in our simulations, albeit with an underestimated value. The effect on cyclopropyl substitution, modeled within the transmembranal groove within TM3-TM6 in pose B, is also captured by the simulations, though the experimental loss in affinity due to the secondary amide of **cmpd 8** is underestimated due to the hydrogen bond modeled with Thr89^3.36^ and His251^6.52^.

Conversely, the aforementioned inner cavity between His251^6.52^ and Thr89^3.36^ binds the substituents on the 6-sulfanyl group when the FEP of the series is simulated on pose A. Such a deep cavity can only accommodate smaller or equally sized groups, which can make polar interactions with this pair of residues in analogy to the modeled carboxamido interactions modeled for BAY60-6583 on the PDB structure 8HDO. While the effect of imidazole replacement (**cmpds 15–19**) seems to be correctly modeled on this pose, the effect of the triazole substitution leads to wrong predictions of increased affinity for **cmpd 20**. Moreover, **cmpd 13** and **cmpd 14**, bearing the bulkiest groups on the 6-sulfanyl substituent, could not be docked on pose A, which is already indicative of a problem on this pose considering the experimental gain in affinity of **cmpd 13**. The hydrophobic cyclopropyl moiety remains solvent exposed, leaning toward Ile276^7.39^, and the replacement by ethoxy in **cmpd 5** leads to higher mobility and disruption of the key hydrogen bond with Asn254^6.55^ and π–π stacking with Phe173^ECL2^ of the core. Considering the above, the results of the FEP simulations of ligand series suggest a more favorable orientation of the scaffold in pose B, in line with the original SAR interpretation of the Colotta group.^46^

The two structures of A_2B_AR in complex with BAY60-6583 were published with abundant site directed mutagenesis (SDM) data, regarding the effect on ligand’s efficacy measured as EC_50_ values from cAMP production assays. Chen et al.^44^ examined the effect of 8 point mutations covering 6 positions in contact with the ligand on their WT structure (7XY6, corresponding to pose B). Five positions were mutated to alanine, two of which (Asn273^7.36^ and Val250^6.51^) were also replaced by the corresponding A_2A_AR side chain (Tyr and Leu, respectively), while Tyr10^1.35^ was only mutated to the most conservative Phe. All mutants showed diminished activation of the A_2B_AR by BAY60-6583, albeit with a varied quantitative range: from the almost negligible effect of the Ser68^2.65^Ala mutant (X-fold EC_50_ = 1.21) to a marked 12.8-fold reduced EC_50_ for Val250^6.51^Leu, defined as a selectivity hotspot between A_2B_ and A_2A_AR for BAY60-6583, in line with our previous report on the role of this mutation on A_2B_AR stereospecific antagonist binding.^52^ On their side, Cai et al.^41^ accompanied the report of the 8HDO structure (pose A) with SDM data corresponding to 20 mutants, including an alanine scan for 18 positions, plus the Ala64^2.61^Ile mutation and the aforementioned Val250^6.51^Leu. Indeed, there is overlapping data for five mutants in the two studies, showing a high degree of correlation between the corresponding experiments (*r*^2^ = 0.88, Figure S12). The effect of all 20 mutants reported by Cai et al.^41^ shows also impairment of BAY60-6583 efficacy, from the negligible effect of Ser68^2.65^ (X-fold EC_50_ = 1.21) to the most pronounced effect of the Val250^6. 51^Leu mutant (X-fold EC_50_ = 105.9).

**Figure 6:**
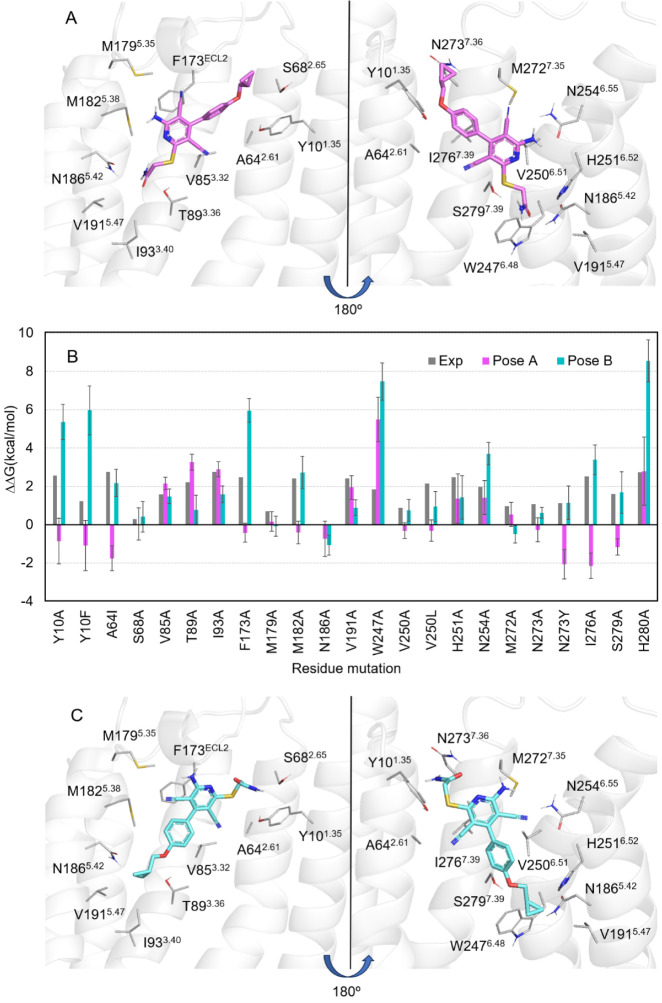
Effect of the point mutations on the BAY60-6583 binding affinity. The binding modes of BAY60-6583 considered are (A) pose A and (C) pose B. (B) Comparison between experimental and calculated RBFE (in kcal·mol^–1^) for A_2B_AR mutants considering the HIP (charged His280^7. 43^) setup for both binding modes (pose A, magenta; pose B, cyan), together with experimental values (gray) as extracted from Cai et al.^41^ and Chen et al.^44^ When two experimental values were available for the same mutation, their arithmetic mean is shown.

In light of these abundant SDM data, we set to perform the corresponding residue FEP simulations to quantify the effect of all 23 mutations on BAY60-6583 affinity. Our QresFEP single-topology protocol of stepwise annihilation is optimal for alanine-scan, which comprises most of the SDM data in these studies. For the remaining mutants, an extra thermodynamic cycle has to be introduced regarding the analogous annihilation of the mutant side chain, which has to be then modeled in a predefined configuration, and the total free energy shift (ΔΔ*G*_mut-WT_) is computed by joining the two thermodynamic cycles.^58^ To minimize the bias induced by the modeling of the “mut” side chain, we took as a reference the rotamer of the corresponding WT position in the A_2A_AR experimental structure (positions 6.51 and 6.55), while assuming the same rotamer for Phe and Tyr (position 1.35). As in the previous part, we performed parallel sets of simulations using either binding mode (i.e., pose A and pose B), in each case considering the alternative protonation states of His280^7.41^ (HID or HIP). The results, expressed as quantitative shifts in binding affinity (ΔΔ*G*_mut-WT_), are presented and compared to experimental data in Table 2, Figures 6, S13, S14, and Tables S2–S5. Initial analysis of this data (extracted from 1.45 μs simulation time, see Section 2 for details on the calculations) shows that the correlation between experimental data and the FEP data is again stronger when the simulations were run on pose B, in particular considering the HIP protonation state. Attending to the standard statistical parameters shown in Table 2, the quantitative correlation strongly favors this pose, showing lower mean unassigned error  = 1.58, , i.e., improved accuracy of 11%), a higher Pearson correlation coefficient ) as well as a better correlation coefficient (, see Figure S14).

As in the ligand SAR analysis, we made the assumption that an experimental shift in EC_50_ directly affects the calculated shift in affinity, which is a general assumption in GPCR agonist design programs but not exempt from quantitative error. This uncertainty in the interpretation of experimental data can add to the error between experiments (see Figure S12) and the known fact that indirect effects of a point mutation can alter the interpretation of SDM data, where FEP studies will mostly capture the direct or at the most short-range indirect effects on ligand binding. Thus, a more useful way of analyzing a large enough pool of in silico SDM data is to determine the predictivity power of the effect of a mutation in a qualitative manner.^98^ In this sense, a mutation can be classified as enhancing, not affecting, or detrimental for the EC_50_ value induced by the reference partial agonist BAY60-6583. We considered a threshold value of ±0.5 kcal·mol^–1^ around the zero value to define these three categories, providing the typical errors on experimental and calculated values. The calculated and experimental values can be easily displayed on a matrix, where the cells are colored according to the qualitative classification of the effect, as shown in Table 2. A semiquantitative Pearson correlation coefficient (PCC*) can be defined on the basis of the three categories established for the mutational effects (Table 2).

Visual inspection of Table 2 and inspection of the associated PCC* values clearly show that the qualitative correlation obtained with pose B_HIP is superior to the other possibilities, revealing itself as the structure that allows the most informative interpretation of the experimental data. Pose B can correctly classify the destabilizing effect of 90% (19 out of 21) mutations and the neutral effect of 1 of the 2 mutations with experimental shift within the ±0.5 kcal·mol^–1^ effect. On the contrary, the sensibility of pose A in reproducing the experimental data is markedly reduced, with only 43% (9/21) of the destabilizing mutations and 1/2 neutral mutations correctly captured, in both cases considering the most predictive HIP setup.

**Table 2:**
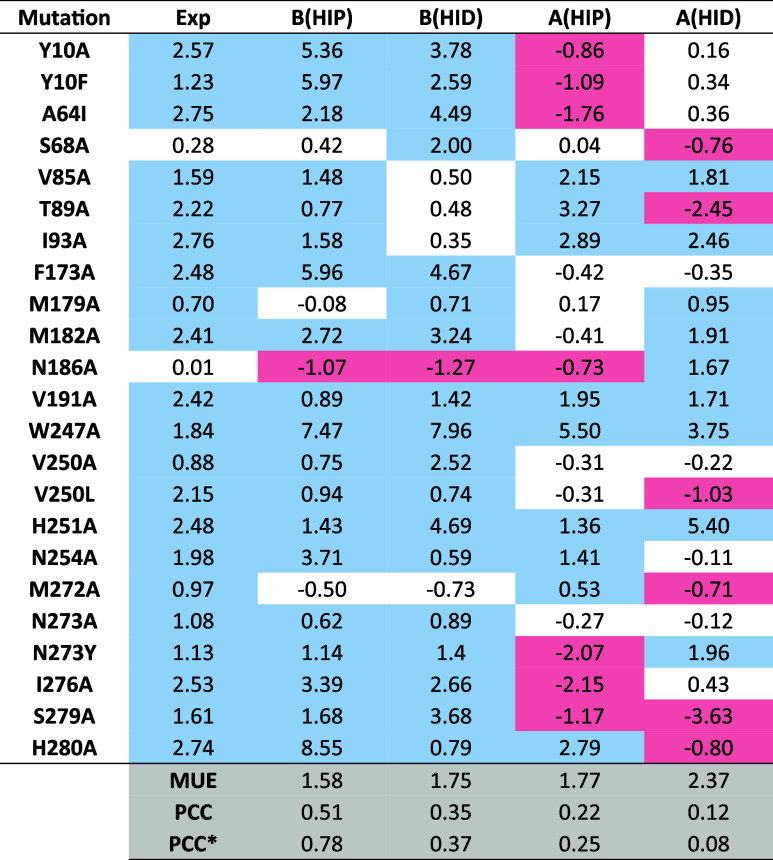
RBFE Shift of BAY60-6583 Due to Single-Point Mutations (ΔΔ*G*_mut-WT_, kcal·mol^–1^) for the 23 SDM Data Points Extracted from the Literature^a^

a The cells are colored according to three categories established for the mutational effects: red (ΔΔ*G* < −0.5 kcal·mol^–1^), white (−0.5 < ΔΔ*G* < 0.5 kcal·mol^–1^), and blue (ΔΔ*G* > 0.5 kcal·mol^–1^). Experimental relative binding free energies (exp) were calculated from EC_50_ values as . Calculated relative binding free energies (ΔΔ*G*_calc_) are obtained from a series of small, convergent FEP calculations  (see Tables S2–S5). MUE: mean unassigned error (kcal·mol^–1^). PCC: Pearson correlation coefficient. PCC*: Pearson correlation coefficient calculated on the three categories established for the mutational effects: −1 (ΔΔ*G* ← 0.5 kcal·mol^–1^), 0 (−0.5 < ΔΔ*G* < 0.5 kcal·mol^–1^), 1 (ΔΔ*G* > 0.5 kcal·mol^–1^).

A few mutational effects are worthwhile to discuss to illustrate the different predictive capabilities of the two models. The most revealing is probably referring to mutations at position Val250^6.51^, a key selectivity hotspot for BAY60-6583^52^ replaced by the bulkier Leu in A_2A_ and remaining AR family members, and the associated data for the corresponding Val250^6.51^Ala mutant. The effect of these mutations is accurately reproduced in pose B, where the ligand is more tightly constrained due to the specific interactions lacking in pose A. Indeed, the simulations show that pose A is more adaptable to the presence of bulkier Leu, in contrast to the experimental data. Likewise, Asn273^7.36^ is a Tyr in the remaining ARs and makes interactions with the acetamide group of BAY60-6583, together with the conserved Tyr10^1.35^, with Ser68^2. 65^ being indirectly involved in the polar network. Consequently, the five SDM data corresponding to these positions are correctly captured by the FEP simulations modeled on pose B, but not so in the corresponding simulations in pose A, where the hydrophobic cyclopropyl is making unspecific hydrophobic interactions.

## 4 Conclusions

The structural revolution of membrane proteins in general and GPCRs in particular has provided coverage for more than 20% of the receptorome, with almost 1,300 class A GPCR structures, from which approximately 50% have been elucidated by cryo-EM techniques. Despite the numerous advantages for mechanistic studies over X-ray crystallography, the resolution of the ligand-binding site of cryo-EM structures is usually lower than that of the X-ray structures. In the case of the A_2B_AR, two cryo-EM structures have been published back-to-back, proposing inverted binding poses for the partial agonist BAY60-6583. In such cases, comparative computational analysis can be the only way to elucidate the physiologically relevant binding mode. We initially performed systematic docking and 1.5 μs MD simulations considering different conditions, the results of which suggest but are not conclusive of one binding orientation, on the basis of geometrical measurements such as ligand or side chain fluctuations or H-bond frequencies. A careful analysis of the available SAR and SDM data in light of accurate, relative binding free energy simulations provides the missing quantitative link between structure and pharmacological data, giving additional insights into the dynamics and specificity of receptor–ligand binding events. In this study, we show the utility of integral analysis of this problem by performing both residue and ligand FEP simulations, providing a comparative evaluation considering alternative binding modes, of the SAR on a series of 10 ligands as well as on the SDM data corresponding to 23 point mutations, which altogether converged in just 1.93 μs of MD sampling. Note that, due to the high computational efficiency of our approach using finite spherical simulations, with relatively short simulation times but allowing 10 replica simulations per ligand or mutation, we could also systematically evaluate the influence on other important parameters, such as the protonation state of a key histidine residue. The results univocally show that binding mode B, proposed on the PDB structure with code 7XY6,^44^ explains well both the SAR and SDM data, particularly if we consider the positively charged state of residue His280^7.43^. One could then wonder why binding mode A has been reported in the cryo-EM structure with PDB code 8HDO.^41^ While this could be due to the wrong assignment of the ligand orientation in the corresponding electron density map, pose A could also correspond to a low probably high energy orientation of BAY60-6583 that is visible under certain experimental conditions. The coclusions of this study can contribute to the design of partial agonist derivatives with improved affinity and selectivity and underscores the value of free energy perturbation methods in structure-based drug design.

## Supporting Information Available

Pairs of topologically equivalent atoms of BAY60-6583; clusters and scores obtained from molecular docking; RMSD, RMSF, and H-bond analyses of NECA and BAY60-6583 complexes with A_2B_AR; RBFE of BAY60-6583 analogues (pose B, HID, pose A, HID and HIP); comparative analysis of experimental affinities of BAY60-6583 due to point mutations; RBFE of BAY60-6583 for A_2B_AR point mutations (HID); calculated vs experimental RBFE of BAY60-6583 for A_2B_AR point mutations (HIP); FEP calculations of BAY60-6583 for A_2B_AR mutants (pose A, pose B, HID, HIP) (PDF)
