# Supplementary material for: Ligand and Residue Free Energy Perturbations Solve the Dual Binding Mode Proposal for an A2BAR Partial Agonist
Source: J Phys Chem B. 2025 Jan 8;129(3):886–99. doi: 10.1021/acs.jpcb.4c07391 (PMC11770768; doi:10.1021/acs.jpcb.4c07391)
Supplement: Supplementary file 1 — jp4c07391_si_001.pdf [file jp4c07391_si_001.pdf]

Supplementary Material for:

Ligand and Residue Free Energy Perturbations Solve the Dual Binding Mode Proposal for a A_{2B}AR partial agonist.

Tana Tandarić[a] and Hugo Gutiérrez-de-Terán[a,*]

[a]Department of Cell and Molecular Biology, Biomedical Center, Uppsala University, Box 590, S-75124 Uppsala, Sweden
[b] Nanomaterials and Nanotechnology Research Center (CINN), Spanish National Research Council (CSIC), Health Research Institute of Asturias (ISPA), Av. del Hospital Universitario, s/n, ES-33011 Oviedo, Asturias, Spain.

Corresponding author: Hugo Gutiérrez-de-Terán, phone +34 985 10 99 05, e-mail: h.g.teran@cinn.es

Contents

S1 ······ Pairs of topologically equivalent atoms of BAY60-6583
S2 ······ Clusters obtained from molecular docking
Table S1 ······ Top docking scores for each cluster
S3 ······ RMSD and RMSF analyses of NECA-A_{2B}AR MD simulations
S4 ······ H-bond analysis of NECA-A_{2B}AR MD simulations
S5 ······ RMSD analysis of BAY60-6583 in complex with A_{2B}AR
S6 ······ RMSD analysis of A_{2B}AR in complex with BAY60-6583
S7 ······ RMSF analysis of A_{2B}AR in complex with BAY60-6583
S8 ······ H-bond analysis of BAY60-6583 in complex with A_{2B}AR
S9 ······ RBFE of BAY60-6583 analogues (pose B, HID)
S10 ······ RBFE of BAY60-6583 analogues (pose A, HIP)
S11 ······ RBFE of BAY60-6583 analogues (pose A, HID)
S12 ······ Comparative analysis of experimental affinities of BAY60-6583 due to point mutations
S13 ······ RBFE of BAY60-6583 for A_{2B}AR point mutations (HID)
S14 ······ Calculated vs Experimental RBFE of BAY60-6583 for A_{2B}AR point mutations (HIP)
Table S2 ······ FEP calculations of BAY60-6583 for A_{2B}AR mutants (pose B, HID)
Table S3 ······ FEP calculations of BAY60-6583 for A_{2B}AR mutants (pose B, HIP)
Table S4 ······ FEP calculations of BAY60-6583 for A_{2B}AR mutants (pose A, HID)
Table S5 ······ FEP calculations of BAY60-6583 for A_{2B}AR mutants (pose A, HIP)

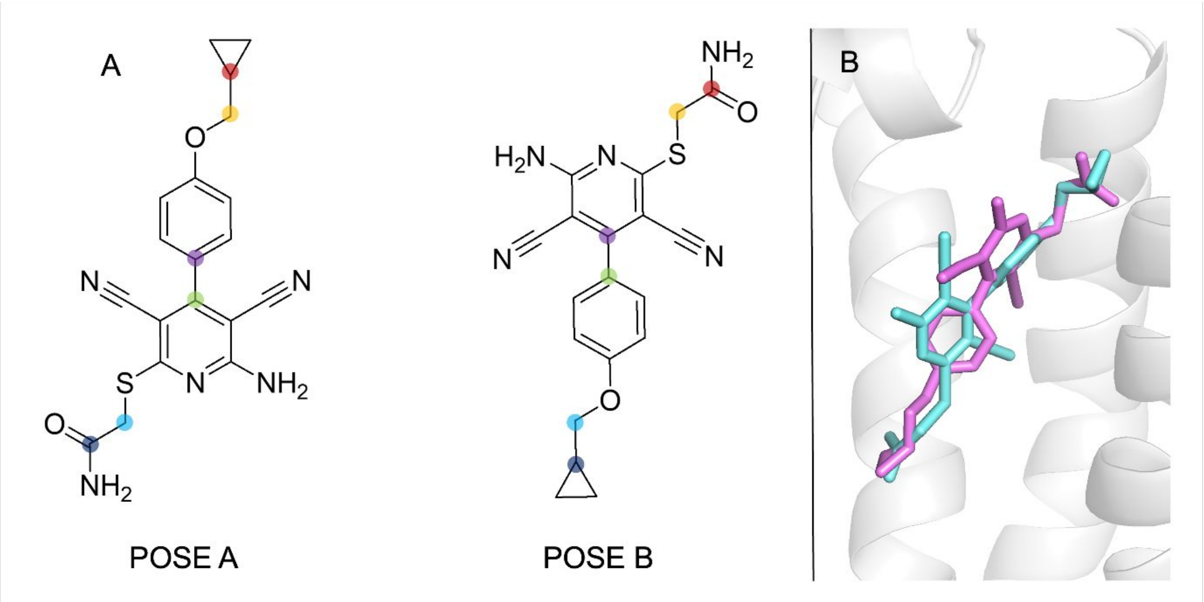

Figure S1. Pairs of topologically equivalent atoms of BAY60-6583 (A) 2D representation of pose A and pose B, related by a flip on the long axis, showing the atoms that lay in equivalent positions with the same color (B) 3D superposition of pose A (cyan) and pose B (magenta). The atoms in equivalent positions are subjected to half-harmonic distance restraints (5.0 kcal/mol/Å²) to maintain each pair within a distance range of 0.0 – 0.2 Å.

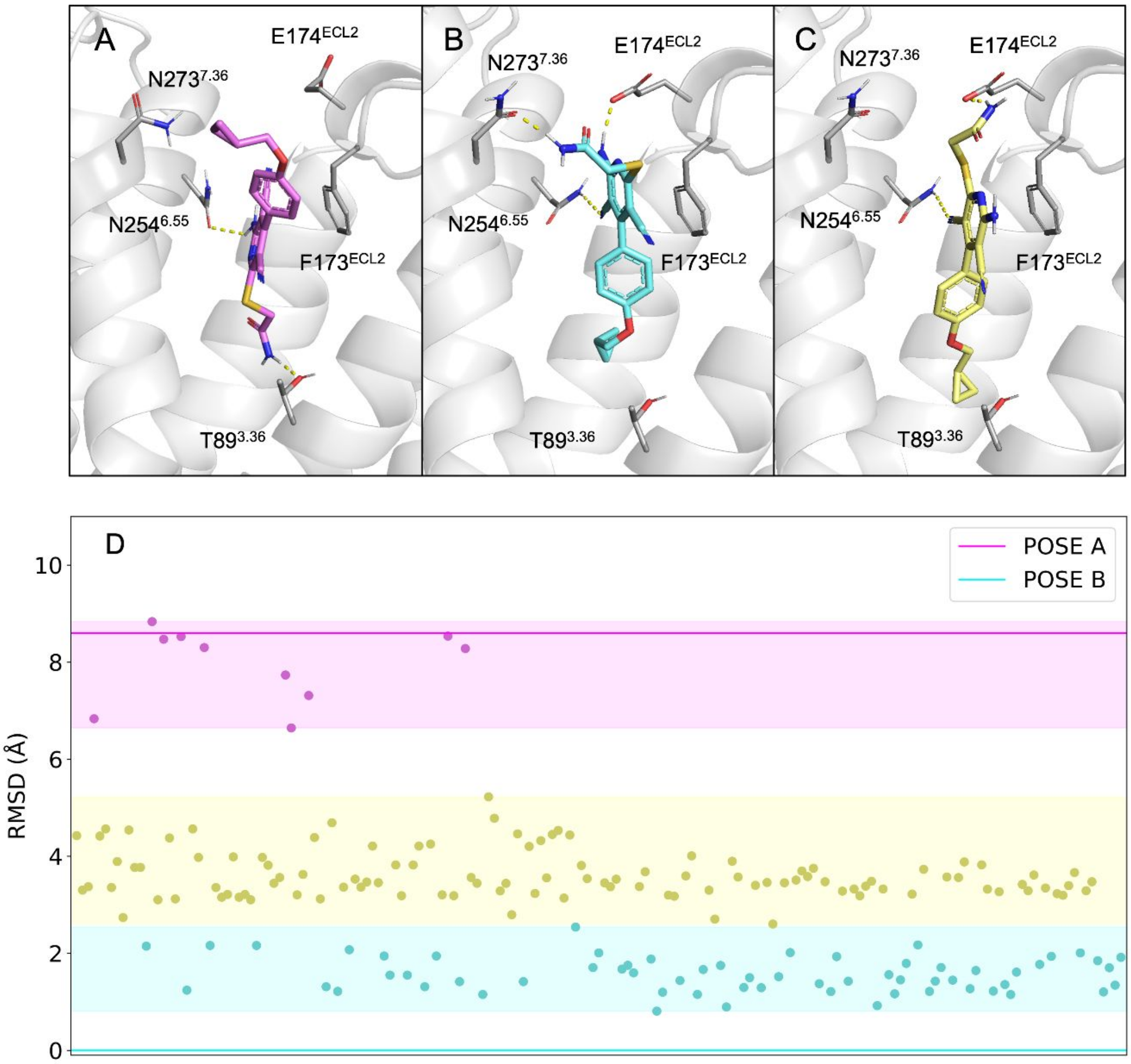

Figure S2. Representative structures of the clusters obtained from molecular docking: (A) Pose A-like orientation, (B) Pose B-like orientation, and (C) Pose B-like orientation with inverted pyridine ring. Hydrogen bonds are shown as yellow dashed lines. (D) RMSD values of the obtained docking poses, with reference orientations of pose A and B marked by lines, and corresponding clusters represented in magenta for cluster A, cyan for cluster B, and yellow for cluster C.

Table S1. Top docking scores (kcal/mol) for each cluster in each protein system

	cluster		
system	A	B	C
8HDO (Hid280$^{7.43}$)	-8.717	-8.623	-8.619
8HDO (Hip280$^{7.43}$)	-8.710	-8.986	-8.623
7XY6 (Hid280$^{7.43}$)	/	-8.831	-7.420
7XY6 (Hip280$^{7.43}$)	/	-8.420	-8.036

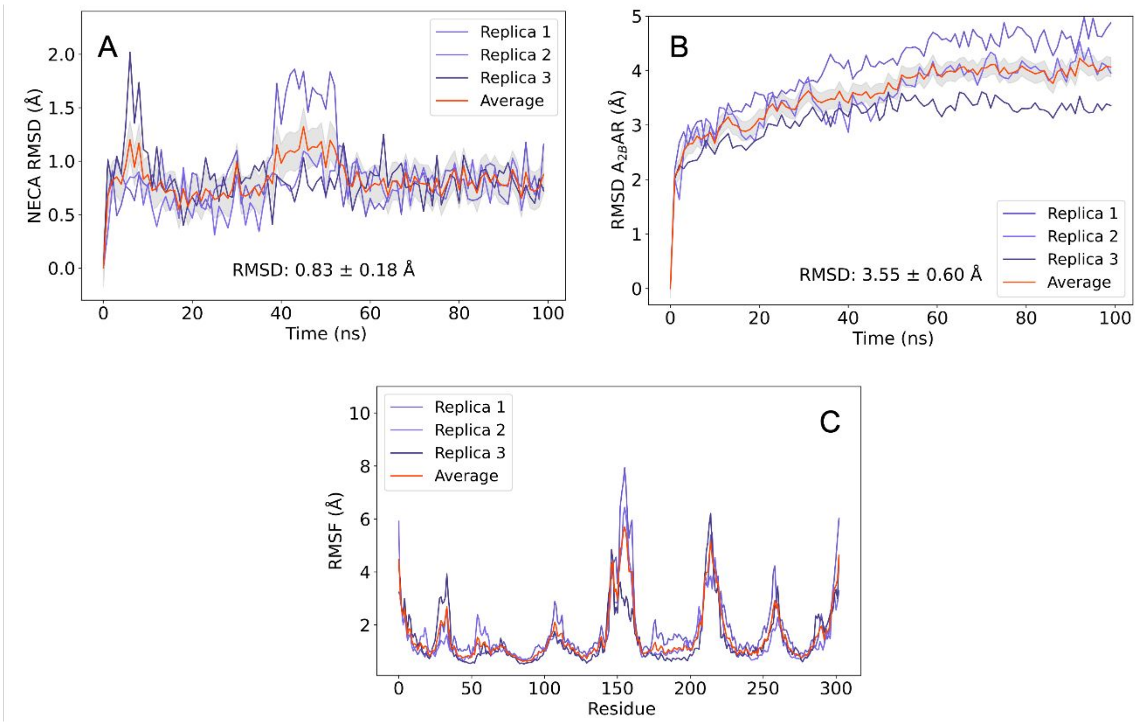

Figure S3. RMSD analysis of NECA-A_{2B}AR MD simulations. (A) NECA RMSD, (B) A_{2B}AR RMSD and (C) RMSF of A_{2B}AR. The average value from triplicate MD simulations is indicated in red, with the grey area in RMSD plots representing the corresponding standard deviation.

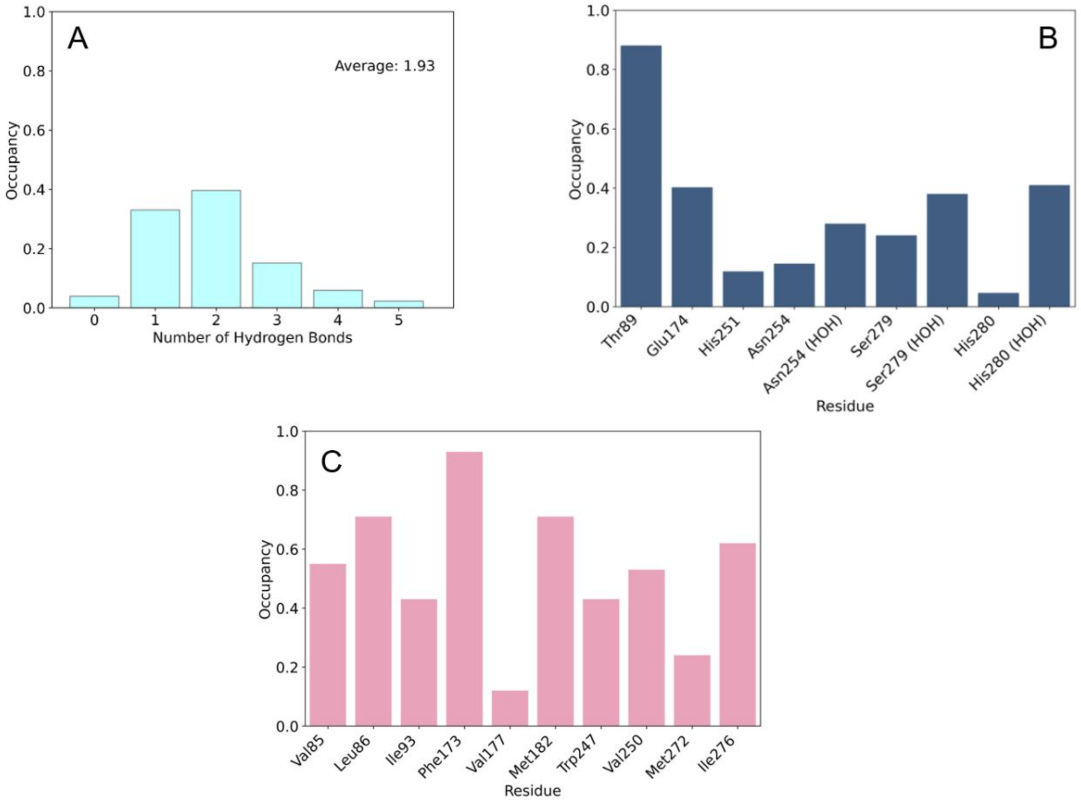

Figure S4. H-bond analysis of NECA-A_{2B}AR MD simulations (A) Number of simultaneous hydrogen bonds between NECA and A_{2B}AR during MD simulations with Hid280$^{7.43}$, using a distance cutoff of 3.5Å and an angle cutoff of 30° (B) Occupancy of hydrogen bonds by residue (included water mediated hydrogen bonds with Asn254$^{6.55}$, Ser279$^{7.42}$ and His280$^{7.43}$) (C) Hydrophobic contact occupancy by residue, defined as hydrophobic sidechains that have any atom within distance d < 3.5 Å of any ligand atom.

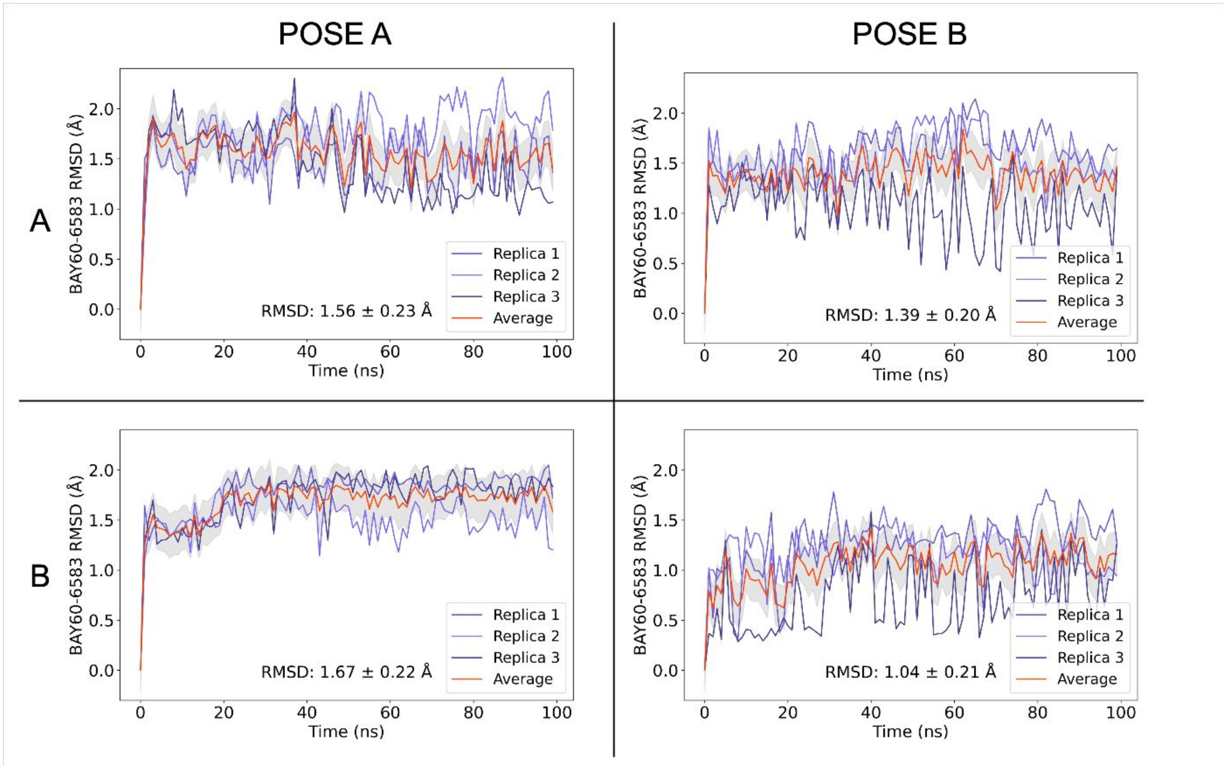

Figure S5. RMSD analysis of BAY60-6583 in complex with $A_{2B}AR$. (A) neutral and (B) protonated His280$^{7.43}$. The average RMSD from triplicate MD simulations is indicated in red, with the grey area representing the corresponding standard deviation.

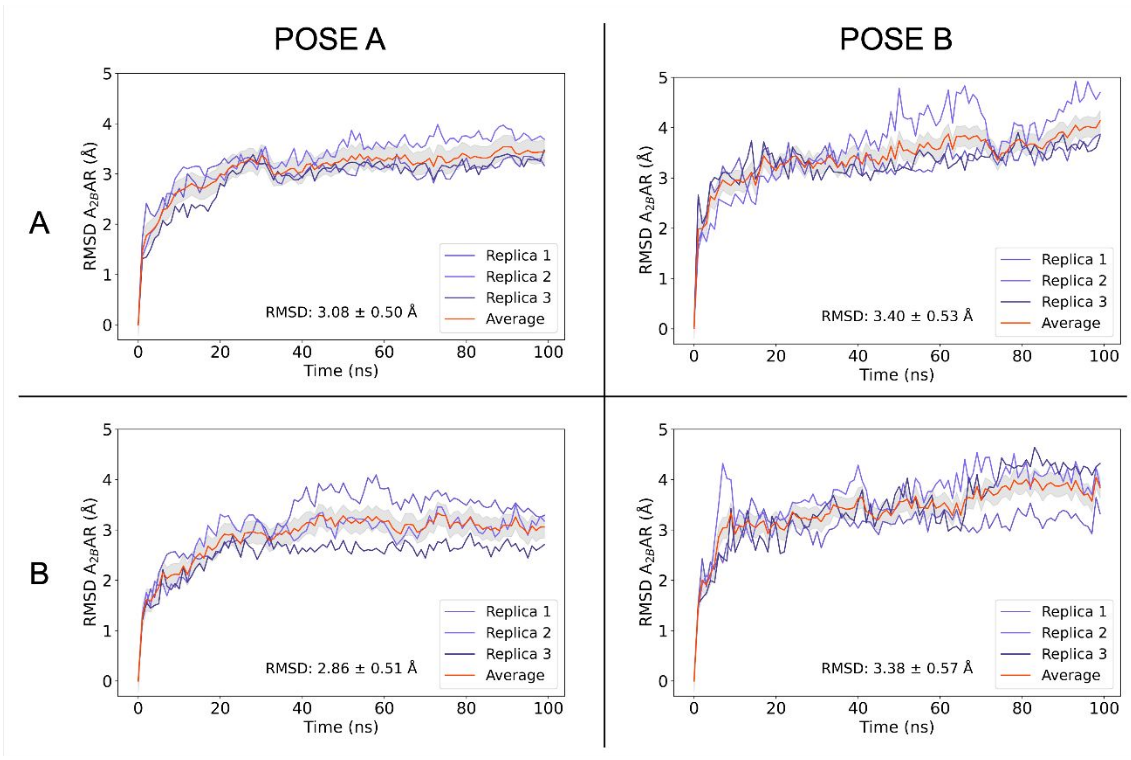

Figure S6. RMSD analysis of $A_{2B}AR$ in complex with BAY60-6583. (A) neutral and (B) protonated His280$^{7.43}$. The average RMSD from triplicate MD simulations is indicated in red, with the grey area representing the corresponding standard deviation.

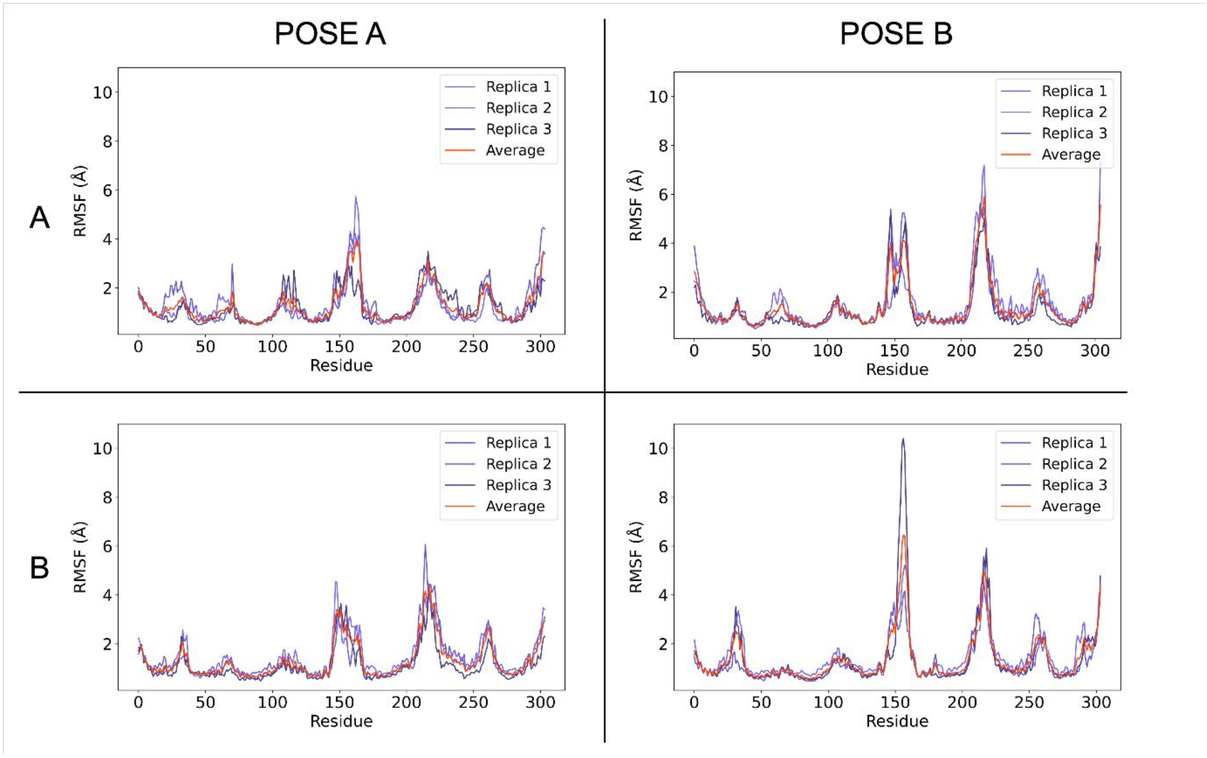

Figure S7. RMSF analysis of $A_{2B}AR$ in complex with BAY60-6583. (A) neutral and (B) protonated His280$^{7.43}$. The average RMSD from triplicate MD simulations is indicated in red, with the grey area representing the corresponding standard deviation.

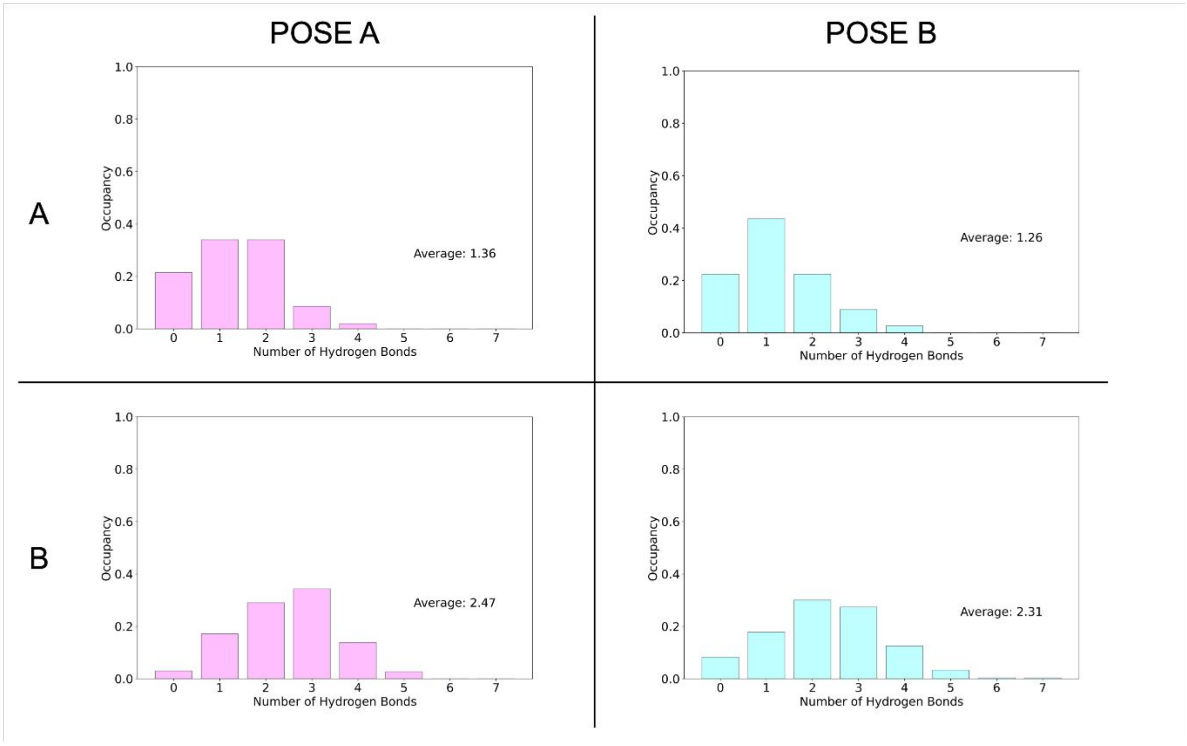

Figure S8. H-bond analysis of BAY60-6583 in complex with BAY60-6583. (A) neutral and (B) protonated His280$^{7.43}$. H-bond is defined using a distance cutoff of 3.5Å and an angle cutoff of 30°.

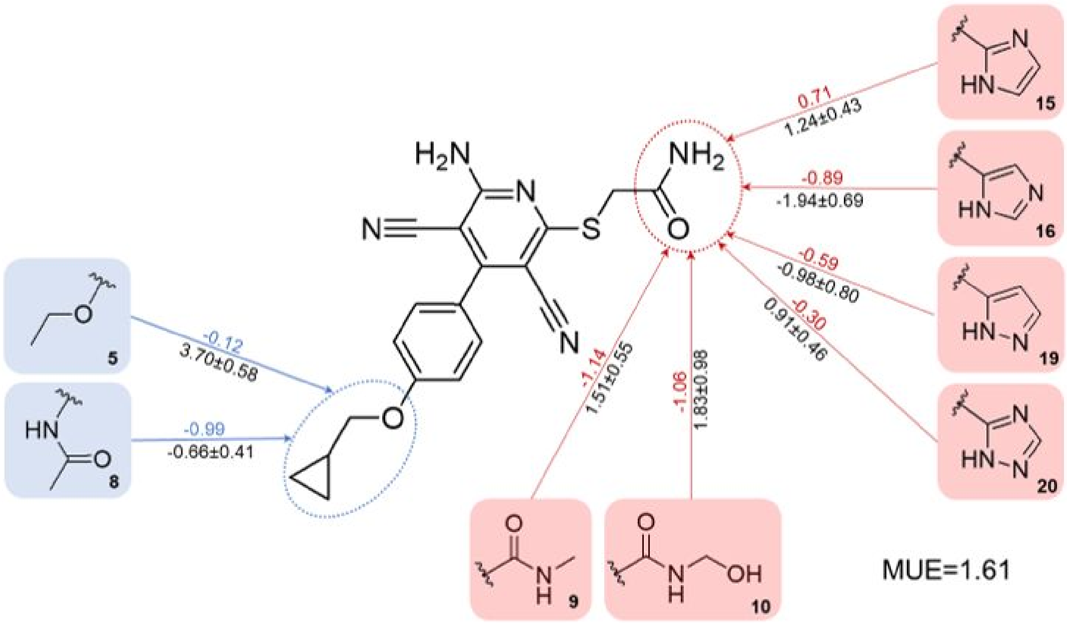

Figure S9. RBFE calculations of one-site analogues with BAY60-6583, run on pose A, in the presence of Hip280$^{7.43}$ (complementary to data presented in main Fig 5). The calculated numbers ($\Delta\Delta G_{L\text{-}BAY}$, kcal·mol^{-1}) follow the direction indicated by the arrow, with FEP values in black (with associated SEM), and experimental values derived from EC_{50} in colored font.

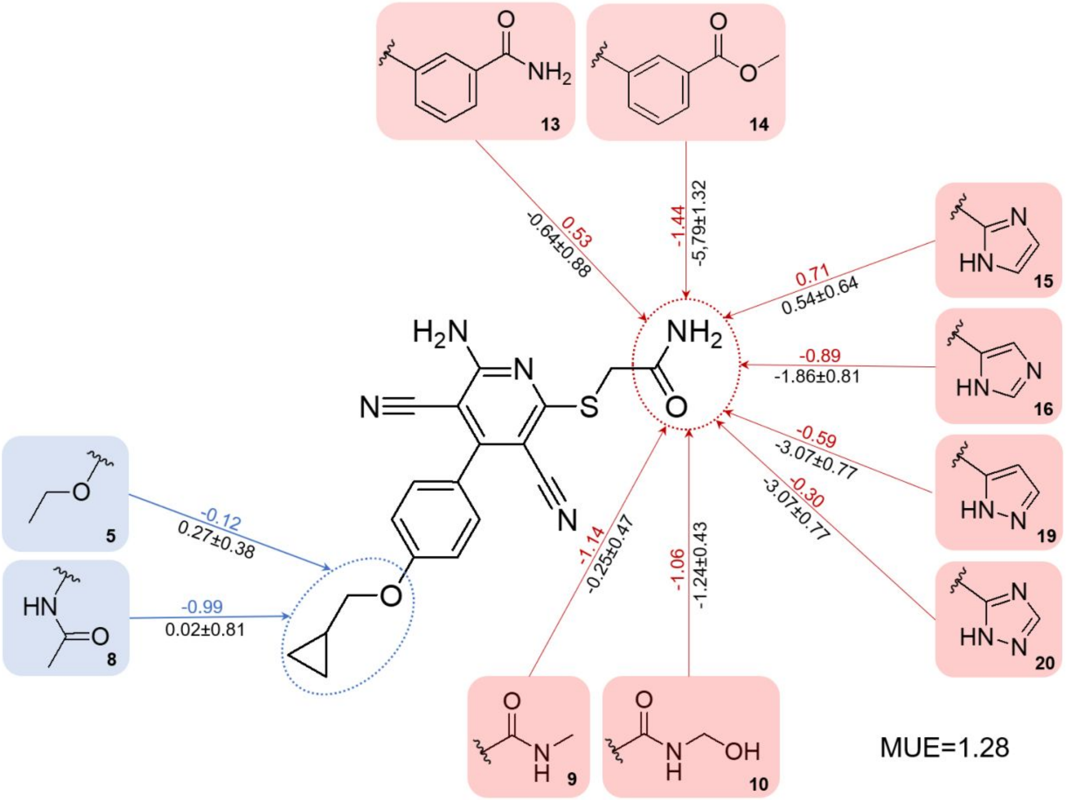

Figure S9. RBFE calculations of one-site analogues with BAY60-6583, run on pose B, in the presence of Hid280[7.43] (complementary to dana presented in main Fig 5). The calculated numbers ($\Delta\Delta G_{L\text{-}BAY}$, kcal·mol^{-1}) follow the direction indicated by the arrow, with FEP values in black (with associated SEM), and experimental values derived from EC_{50} in colored font.

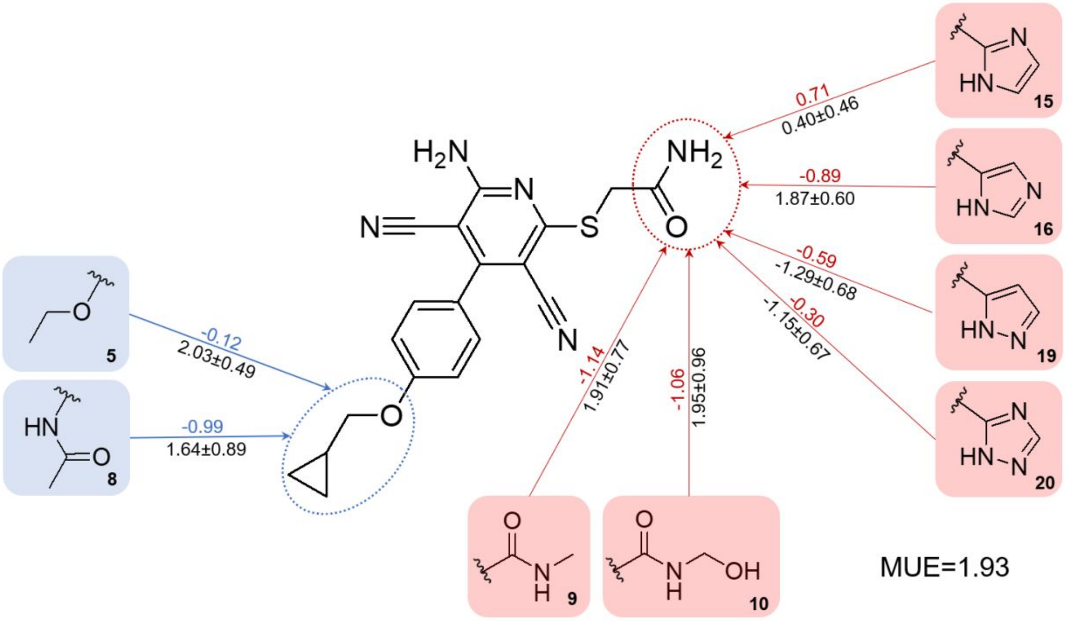

Figure S11. RBFE calculations of one-site analogues with BAY60-6583, run on pose A, in the presence of Hid280[7.43] (complementary to data presented in main Fig 5). The calculated numbers ($\Delta\Delta G_{L\text{-}BAY}$, kcal·mol^{-1}) follow the direction indicated by the arrow, with FEP values in black (with associated SEM), and experimental values derived from EC_{50} in colored font.

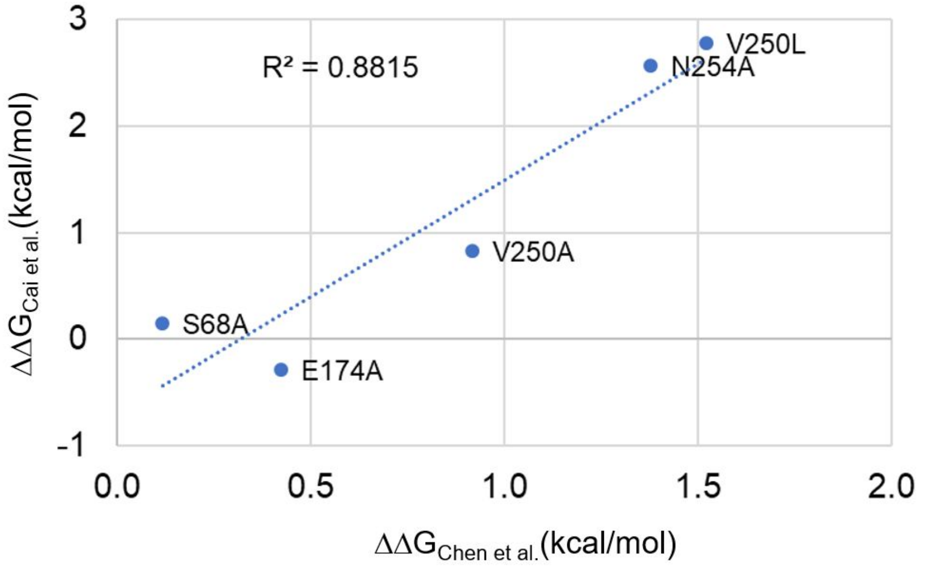

Figure S12. Comparative analysis of experimental affinities of BAY60-6583 due to point mutations. Data is expressed as $\Delta\Delta G$ (kcal·mol^{-1}) extracted from experimental EC_{50} values reported by Cai et al. (Y axis) or by Chen et al (X axis). The correlation coefficient is shown in the figure, along with the regression line.

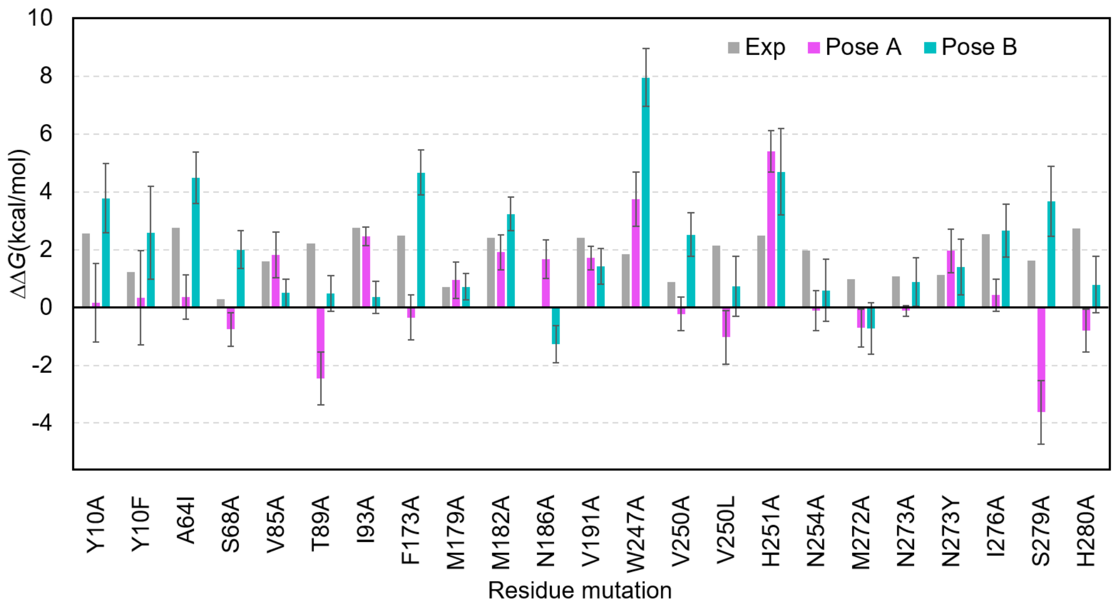

Figure S13. RBFE (kcal·mol^{-1}) of BAY60-6583 due to $A_{2B}AR$ point mutations considering HID (neutral His280$^{7.43}$) setup. Both binding modes (pose A, magenta; pose B, cyan) are plotted together with experimental values (gray) as extracted from Cai et al and Chen et al. When two experimental values were available for the same mutation, their arithmetic mean is shown.

Figure S14. Calculated vs Experimental RBFE of BAY60-6583 for 23 A_{2B}AR point mutations ($\Delta\Delta G_{\text{Mut-WT}}$, kcal·mol^{-1}), considering the HIP (protonated His280$^{7.43}$) setup. (Left) pose A, (Right) pose B. Experimental values obtained as in Supplementary Figure S13.

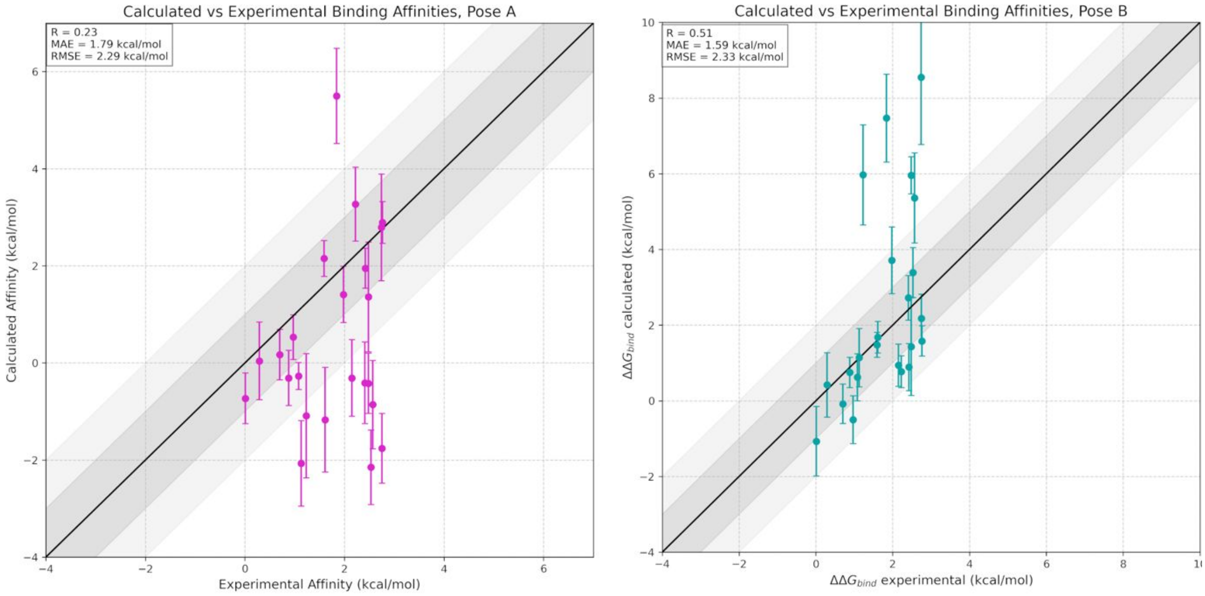

Table S2. FEP calculations (in kcal·mol-1) of BAY60-6583 for A_{2B}AR mutants (pose B, modelled based on the 7XY6 structure, HID, neutral His280$^{7.43}$), together with the corresponding experimental values.

WT-sidechain	*X*	$\Delta\Delta G_{holo}$	$\Delta\Delta G_{apo}$	$\Delta\Delta G_{FEP1}$	$\Delta\Delta G_{FEP2}$	$\Delta\Delta G_{FEP3}$	$\Delta\Delta G_{FEP4}$	$\Delta\Delta G_{FEP5}$	$\Delta\Delta G_{FEP6}$	$\Delta\Delta G_{FEP7}$	$\Delta\Delta G_{FEP8}$	$\Delta\Delta G_{FEP9}$	$\Delta\Delta G_{calc}$ *(X→Ala)*	$\Delta\Delta G_{calc}$ *(WT→X)*	$\Delta\Delta G_{exp}$ *(Chen et al)*	$\Delta\Delta G_{exp}$ *(Cai et al)*
Tyr10$^{1.35}$	Tyr	9.7±1.1	5.9±0.6	4.5±0.7	0.0±0.1	0.7±0.5	-0.6±0.4	-0.7±0.8	-0.4±0.2	0.3±0.3	0.0±0.0		3.8±1.2		ND[b]	2.6
	Phe	-6.6±0.8	-7.8±0.8	0.2±0.1	-0.1±0.1	0.7±0.3	0.5±0.2	-0.1±0.9	-0.1±0.4	0.0±0.4	0.0±0.0		1.2±1.1	2.6±1.6	1.2	ND
Ala64$^{2.61}$	Ile	-16.8±0.8	-12.3±0.4	0.3±0.1	-0.5±0.1	-1.3±0.2	-2.4±0.3	-0.6±0.3	0.0±0.0				-4.5±0.9	4.5±0.9	ND	2.8
Ser68$^{2.65}$	Ser	-5.7±0.4	-7.7±0.5	1.6±0.5	-0.2±0.1	0.6±0.1	-0.1±0.0						2.0±0.7		0.4	0.2
Val85$^{3.32}$	Val	-5.3±0.4	-5.8±0.3	-0.3±0.2	-0.2±0.1	0.2±0.4	0.9±0.3	-0.1±0.0					0.5±0.5		ND	1.6
Thr89$^{3.36}$	Thr	-0.9±0.5	-1.3±0.3	-1.1±0.2	0.2±0.1	0.6±0.3	0.9±0.3	-0.1±0.0					0.5±0.6		ND	2.2
Ile93$^{3.40}$	Ile	-6.4±0.3	-6.7±0.5	0.0±0.0	-0.2±0.1	0.0±0.1	-1.0±0.3	1.4±0.4	0.1±0.0				0.4±0.6		ND	2.8
Phe173^{ECL2}	Phe	0.4±0.8	-4.2±0.2	-0.2±0.1	0.2±0.1	0.1±0.1	0.4±0.2	2.2±0.4	1.3±0.3	0.5±0.2	0.0±0.0		4.7±0.8		ND	2.5
Met179$^{5.35}$	Met	-1.1±0.3	-1.8±0.4	0.2±0.1	-0.1±0.0	0.2±0.2	0.0±0.2	0.4±0.2	0.0±0.2	0.0±0.0			0.7±0.5		ND	0.7
Met182$^{5.38}$	Met	5.4±0.5	2.2±0.3	0.1±0.3	0.0±0.0	0.0±0.2	0.6±0.1	1.8±0.2	0.7±0.3	0.0±0.0			3.2±0.6		ND	2.4
Asn186$^{5.42}$	Asn	36.9±0.6	38.2±0.3	-1.1±0.7	-0.5±0.1	0.0±0.2	0.2±0.3	0.1±0.0					-1.3±0.6		ND	0.0
Val191$^{5.47}$	Val	-7.4±0.6	-8.8±0.3	-0.1±0.1	-0.1±0.1	0.0±0.3	1.6±0.4	0.0±0.0					1.4±0.6		ND	2.4
Trp247$^{6.48}$	Trp	14.7±0.9	6.7±0.5	-0.2±0.1	0.2±0.1	0.4±0.2	0.7±0.3	3.0±0.4	1.9±0.5	1.1±0.3	0.8±0.3	0.1±0.0	8.0±1.0		ND	1.8
Val250$^{6.51}$	Val	-5.0±0.7	-7.5±0.4	-0.2±0.1	0.0±0.1	0.8±0.6	2.0±0.1	-0.1±0.6					2.5±0.8		0.9	0.8
	Leu	-4.5±0.6	-6.3±0.4	0.0±0.1	-0.4±0.1	0.6±0.3	0.1±0.6	1.5±0.1	-0.1±0.1				1.8±0.7	0.7±1.0	1.5	2.8
His251$^{6.52}$	His	20.9±1.4	16.2±0.6	1.2±1.4	0.0±0.0	0.4±0.2	0.8±0.3	1.5±0.5	0.9±0.3	-0.1±0.0			4.7±1.5		ND	2.5
Asn254$^{6.55}$	Asn	36.3±0.8	35.7±0.7	-0.4±1.1	-0.2±0.1	0.6±0.1	0.6±0.3	0.0±0.0					0.6±1.1		1.4	2.6
Met272$^{7.35}$	Met	-1.2±0.7	-0.4±0.6	-0.6±0.4	0.0±0.0	0.1±0.3	-0.2±0.3	0.0±0.4	0.0±0.3	0.0±0.0			-0.7±0.9		ND	1.0
Asn273$^{7.36}$	Asn	35.4±0.5	34.5±0.7	0.4±0.7	0.2±0.1	0.7±0.2	-0.3±0.2	-0.1±0.0					0.9±0.8		1.1	ND
	Tyr	8.1±0.2	8.6±0.4	0.3±0.0	0.2±0.1	0.2±0.3	0.4±0.3	-0.6±0.5	-1.1±0.1	0.1±0.1	0.0±0.0		-0.5±0.5	1.4±1.0	1.1	ND
Ile276$^{7.39}$	Ile	-10.7±0.8	-13.4±0.5	-0.2±0.1	0.1±0.1	0.3±0.1	1.5±0.5	0.8±0.5	0.1±0.0				2.7±0.9		ND	2.5
Ser279$^{7.42}$	Ser	-4.4±0.4	-8.1±1.2	3.1±1.3	0.4±0.0	0.4±0.2	-0.1±0.0						3.7±1.2		ND	1.6
His280$^{7.43}$	His	13.9±0.7	13.1±0.7	-5.9±0.9	0.1±0.0	1.0±0.1	2.6±0.5	1.8±0.3	1.2±0.2	0.0±0.0			0.8±1.0		ND	2.7

[a] *Experimental relative binding free energies* ($\Delta\Delta G_{exp}$) *were calculated from* EC_{50} *values as* $\Delta\Delta G_{exp} = RTln(\frac{EC_{50}^{mut}}{EC_{50}^{wt}})$. *Calculated relative binding free energies* ($\Delta\Delta G_{calc}$) *are obtained from series of small, convergent FEP calculations* $((\Delta G_{holo}^{wt} - \Delta G_{apo}^{wt}) - (\Delta G_{holo}^{mut} - \Delta G_{apo}^{mut}))$

[b] *ND: not determined*

Table S3. FEP calculations (in kcal·mol-1) of BAY60-6583 for $A_{2B}AR$ mutants (pose B, modelled based on the 7XY6 structure, HIP, positively charged His280$^{7.43}$), together with the corresponding experimental values.

WT-sidechain	*X*	$\Delta\Delta G_{holo}$	$\Delta\Delta G_{apo}$	$\Delta\Delta G_{FEP1}$	$\Delta\Delta G_{FEP2}$	$\Delta\Delta G_{FEP3}$	$\Delta\Delta G_{FEP4}$	$\Delta\Delta G_{FEP5}$	$\Delta\Delta G_{FEP6}$	$\Delta\Delta G_{FEP7}$	$\Delta\Delta G_{FEP8}$	$\Delta\Delta G_{FEP9}$	$\Delta\Delta G_{calc}$ *(X→Ala)*	$\Delta\Delta G_{calc}$ *(WT→X)*	$\Delta\Delta G_{exp}$ *(Chen et al)*	$\Delta\Delta G_{exp}$ *(Cai et al)*
Tyr10$^{1.35}$	Tyr	11.2±0.6	5.8±1.0	5.3±0.8	0.4±0.1	0.9±0.4	-0.1±0.2	-0.7±0.4	-0.8±0.4	0.4±0.2	0.1±0.0		5.4±1.2		ND	2.6
	Phe	-8.6±0.4	-8.0±0.4	0.4±0.1	0.1±0.1	0.0±0.2	0.4±0.1	-0.6±0.6	-1.2±0.3	0.2±0.1	0.0±0.0		-0.6±0.6	6.0±1.3	1.2	ND
Ala64$^{2.61}$	Ile	-13.5±0.6	-11.3±0.3	-0.2±0.0	-0.2±0.1	-1.1±0.2	-1.3±0.3	0.6±0.2	0.0±0.0				-2.2±0.6	2.2±0.6	ND	2.8
Ser68$^{2.65}$	Ser	-7.3±0.6	-7.7±0.6	0.4±0.9	0.3±0.0	-0.3±0.1	0.0±0.0						0.4±0.8		0.4	0.2
Val85$^{3.32}$	Val	-4.5±0.2	-6.0±0.2	-0.3±0.2	-0.1±0.0	0.6±0.2	1.0±0.3	0.2±0.0					1.5±0.3		ND	1.6
Thr89$^{3.36}$	Thr	-1.5±0.2	-2.3±0.3	1.4±0.4	-0.2±0.1	0.0±0.2	-0.5±0.3	0.0±0.0					0.8±0.4		ND	2.2
Ile93$^{3.40}$	Ile	-3.8±0.3	-5.3±0.3	0.1±0.0	-0.4±0.1	-0.7±0.1	1.3±0.3	1.2±0.3	0.1±0.0				1.6±0.4		ND	2.8
Phe173^{ECL2}	Phe	1.0±0.4	-4.9±0.2	0.2±0.1	0.2±0.0	0.2±0.1	0.8±0.1	2.6±0.2	1.8±0.3	0.2±0.1	0.0±0.0		6.0±0.5		ND	2.5
Met179$^{5.35}$	Met	-2.2±0.4	-2.2±0.3	-0.3±0.2	0.0±0.0	0.3±0.3	-0.1±0.3	-0.4±0.4	0.3±0.1	0.0±0.0			-0.1±0.5		ND	0.7
Met182$^{5.38}$	Met	5.5±0.4	2.8±0.4	-0.2±0.2	0.0±0.0	0.6±0.2	0.4±0.2	1.4±0.3	0.5±0.2	0.0±0.0			2.7±0.6		ND	2.4
Asn186$^{5.42}$	Asn	36.8±0.6	37.9±0.7	-1.7±0.9	-0.6±0.1	0.2±0.2	1.0±0.2	0.1±0.0					-1.1±0.9		ND	0.0
Val191$^{5.47}$	Val	-6.8±0.6	-7.6±0.3	-0.1±0.0	0.0±0.1	0.1±0.3	0.9±0.3	0.0±0.0					0.9±0.6		ND	2.4
Trp247$^{6.48}$	Trp	14.1±0.8	6.6±0.9	-0.4±0.1	0.1±0.1	0.3±0.1	0.8±0.2	2.0±0.4	1.7±0.5	1.8±0.3	1.1±0.1	0.1±0.0	7.5±1.2		ND	1.8
Val250$^{6.51}$	Val	-5.1±0.3	-5.8±0.3	-0.4±0.2	0.0±0.1	-0.1±0.2	1.2±0.2	-0.1±0.1					0.8±0.4		0.9	0.8
	Leu	-4.7±0.3	-4.6±0.3	0.0±0.0	0.1±0.1	-0.1±0.2	0.2±0.6	-0.5±0.2	0.1±0.0				-0.2±0.4	0.9±0.6	1.5	2.8
His251$^{6.52}$	His	19.3±1.1	17.8±0.7	-1.3±0.8	0.0±0.0	0.1±0.1	0.2±0.4	1.9±0.5	0.7±0.3	-0.1±0.0			1.4±1.3		ND	2.5
Asn254$^{6.55}$	Asn	38.1±0.6	34.4±0.7	2.2±1.0	-0.2±0.1	0.7±0.2	0.9±0.3	0.0±0.0					3.7±0.9		1.4	2.6
Met272$^{7.35}$	Met	-1.5±0.4	-1.0±0.5	-0.6±0.3	0.0±0.0	0.1±0.1	-0.4±0.2	0.2±0.3	0.2±0.1	0.0±0.0			-0.5±0.6		ND	1.0
Asn273$^{7.36}$	Asn	34.9±0.5	34.3±0.4	0.4±0.7	0.2±0.1	0.6±0.2	-0.4±0.2	-0.1±0.0					0.6±0.6		1.1	ND
	Tyr	7.9±0.4	8.5±0.2	0.3±0.0	0.2±0.1	0.0±0.1	0.5±0.1	-0.5±0.5	-1.2±0.3	0.1±0.1	0.1±0.0		-0.5±0.5	1.1±0.8	1.1	ND
Ile276$^{7.39}$	Ile	-7.4±0.6	-10.8±0.3	0.1±0.1	-0.3±0.1	0.7±0.2	1.3±0.4	1.5±0.3	0.0±0.0				3.4±0.7		ND	2.5
Ser279$^{7.42}$	Ser	-4.2±0.4	-5.9±0.2	1.0±0.3	0.5±0.1	0.5±0.3	-0.3±0.0						1.7±0.4		ND	1.6
His280$^{7.43}$	Hip	74.4±1.3	65.9±1.2	5.4±1.1	0.1±0.0	1.0±0.2	1.1±0.1	1.3±0.3	0.6±0.3	0.0±0.0			8.55±1.8		ND	2.7

[a] *Experimental relative binding free energies ($\Delta\Delta G_{exp}$) were calculated from EC_{50} values as* $\Delta\Delta G_{exp} = RTln(\frac{EC_{50}^{mut}}{EC_{50}^{wt}})$ *. Calculated relative binding free energies ($\Delta\Delta G_{calc}$) are obtained from series of small, convergent FEP calculations* $((\Delta G_{holo}^{wt} - \Delta G_{apo}^{wt}) - (\Delta G_{holo}^{mut} - \Delta G_{apo}^{mut}))$

[b] *ND: not determined*

Table S4. FEP calculations (in kcal·mol-1) of BAY60-6583 for A_{2B}AR mutants (pose A, modelled based on the 8HDO structure, HID, neutral His280$^{7.43}$), together with the corresponding experimental values.

WT-sidechain	*X*	$\Delta\Delta G_{holc}$	$\Delta\Delta G_{apo}$	$\Delta\Delta G_{FEP1}$	$\Delta\Delta G_{FEP2}$	$\Delta\Delta G_{FEP3}$	$\Delta\Delta G_{FEP4}$	$\Delta\Delta G_{FEP5}$	$\Delta\Delta G_{FEP6}$	$\Delta\Delta G_{FEP7}$	$\Delta\Delta G_{FEP8}$	$\Delta\Delta G_{FEP9}$	$\Delta\Delta G_{calc}$ *(X→ Ala)*	$\Delta\Delta G_{calc}$ *(WT→ X)*	$\Delta\Delta G_{exp}$ *(Chen et al)*	$\Delta\Delta G_{exp}$ *(Cai et al)*
Tyr10$^{1.35}$	Tyr	3.8±1.1	3.6±0.9	0.5±0.5	-0.2±0.1	0.6±0.4	-0.1±0.3	-0.2±0.6	-0.8±0.2	0.3±0.3	0.0±0.0		0.2±1.4		ND	2.6
	Phe	-8.7±0.7	-8.5±0.5	-0.2±0.1	-0.6±0.2	0.7±0.2	0.5±0.4	0.0±0.7	-0.6±0.2	-0.1±0.3	0.0±0.0		-0.2±0.9	0.3±1.6	1.2	ND
Ala64$^{2.61}$	Ile	-13.9±0.6	-13.5±0.4	0.2±0.1	-0.4±0.1	0.0±0.2	-0.3±0.3	0.3±0.4	-0.1±0.0				-0.4±0.8	0.4±0.8	ND	2.8
Ser68$^{2.65}$	Ser	-7.5±0.3	-6.8±0.5	-0.3±0.6	-0.5±0.1	0.1±0.1	-0.1±0.0						-0.8±0.6		0.4	0.2
Val85$^{3.32}$	Val	-6.7±0.5	-8.5±0.6	0.0±0.1	-0.4±0.1	0.4±0.3	1.8±0.5	-0.1±0.0					1.8±0.8		ND	1.6
Thr89$^{3.36}$	Thr	-2.9±0.8	-0.4±0.5	0.6±0.3	-0.2±0.1	-1.3±0.4	-1.4±0.6	-0.1±0.0					-2.5±0.9		ND	2.2
Ile93$^{3.40}$	Ile	-2.1±0.2	-4.5±0.2	0.0±0.0	0.1±0.0	0.6±0.2	0.3±0.2	1.3±0.3	0.1±0.0				2.5±0.3		ND	2.8
Phe173^{ECL2}	Phe	-5.1±0.8	-4.8±0.2	0.1±0.1	0.1±0.1	-0.2±0.2	0.2±0.2	-1.0±0.3	-0.1±0.3	0.5±0.2	0.1±0.1		-0.4±0.8		ND	2.5
Met179$^{5.35}$	Met	-0.1±0.5	-1.1±0.4	0.2±0.2	0.0±0.0	0.5±0.1	0.2±0.3	-0.1±0.3	0.2±0.3	0.0±0.0			1.0±0.6		ND	0.7
Met182$^{5.38}$	Met	4.1±0.6	2.2±0.3	-0.5±0.3	0.0±0.0	1.0±0.2	-0.5±0.2	1.3±0.3	0.5±0.2	0.0±0.0			1.9±0.6		ND	2.4
Asn186$^{5.42}$	Asn	38.8±0.7	37.1±0.2	1.3±0.5	-0.3±0.1	-0.2±0.1	0.8±0.2	0.0±0.0					1.7±0.7		ND	0.0
Val191$^{5.47}$	Val	-3.2±0.2	-4.9±0.4	0.1±0.0	-0.2±0.0	0.1±0.1	1.7±0.2	0.0±0.0					1.7±0.4		ND	2.4
Trp247$^{6.48}$	Trp	13.7±0.6	9.9±0.7	-0.3±0.1	-0.3±0.0	0.3±0.3	-2.6±1.0	2.1±0.4	1.8±1.4	1.8±0.4	1.1±0.2	0.0±0.1	3.8±0.9		ND	1.8
Val250$^{6.51}$	Val	-8.3±0.4	-8.1±0.4	0.5±0.4	-0.1±0.1	-0.8±0.5	0.2±0.3	0.0±0.1					-0.2±0.6		0.9	0.8
	Leu	-7.1±0.4	-7.9±0.6	-0.2±0.0	-0.4±0.1	0.5±0.1	0.5±0.4	0.3±0.2	0.0±0.0				0.8±0.7	-1.0±0.9	1.5	2.8
His251$^{6.52}$	His	21.3±0.5	15.9±0.6	2.8±0.4	0.0±0.0	-0.1±0.1	0.1±0.3	1.4±0.3	1.3±0.2	-0.1±0.0			5.4±0.7		ND	2.5
Asn254$^{6.55}$	Asn	37.5±0.4	37.6±0.5	0.2±0.6	0.1±0.1	-0.1±0.3	-0.3±0.5	0.0±0.0					-0.1±0.7		1.4	2.6
Met272$^{7.35}$	Met	-1.9±0.4	-1.1±0.5	0.3±0.2	0.0±0.0	0.1±0.1	-0.7±0.1	-0.4±0.3	0.0±0.2	0.0±0.0			-0.7±0.7		ND	1.0
Asn273$^{7.36}$	Asn	33.6±0.1	33.7±0.2	0.2±0.3	-0.2±0.1	-0.3±0.1	0.2±0.1	0.0±0.0					-0.1±0.2		1.1	ND
	Tyr	0.7±0.4	2.7±0.6	0.0±0.5	-0.3±0.2	-0.4±0.2	-0.3±0.2	-1.0±0.4	0.0±0.2	-0.1±0.3	0.0±0.0		-2.1±0.7	2.0±0.8	1.1	ND
Ile276$^{7.39}$	Ile	-11.3±0.5	-11.7±0.3	0.1±0.0	-0.2±0.1	0.0±0.2	0.2±0.3	0.1±0.3	0.2±0.0				0.4±0.6		ND	2.5
Ser279$^{7.42}$	Ser	-6.7±0.9	-3.1±0.6	-4.0±1.4	-0.2±0.0	0.4±0.1	0.1±0.0						-3.6±1.1		ND	1.6
His280$^{7.43}$	His	14.5±0.7	15.3±0.3	-5.2±0.3	0.0±0.0	0.6±0.1	2.1±0.4	0.6±0.2	1.1±0.3	0.1±0.0			-0.8±0.7		ND	2.7

[a] *Experimental relative binding free energies* ($\Delta\Delta G_{exp}$) *were calculated from* EC_{50} *values as* $\Delta\Delta G_{exp} = RTln(\frac{EC_{50}^{mut}}{EC_{50}^{wt}})$. *Calculated relative binding free energies* ($\Delta\Delta G_{calc}$) *are obtained from series of small, convergent FEP calculations* $((\Delta G_{holo}^{wt} - \Delta G_{apo}^{wt}) - (\Delta G_{holo}^{mut} - \Delta G_{apo}^{mut}))$

[b] *ND: not determine*

Table S5. FEP calculations (in kcal·mol-1) of BAY60-6583 for A_{2B}AR mutants (pose A, modelled based on the 8HDO structure, HIP, positively charged His280$^{7.43}$), together with the corresponding experimental values.

WT-sidechain	*X*	$\Delta\Delta G_{holo}$	$\Delta\Delta G_{apo}$	$\Delta\Delta G_{[illegible]}$	$\Delta\Delta G_{FEP2}$	$\Delta\Delta G_{FEP3}$	$\Delta\Delta G_{FEP4}$	$\Delta\Delta G_{FEP5}$	$\Delta\Delta G_{FEP6}$	$\Delta\Delta G_{FEP7}$	$\Delta\Delta G_{FEP8}$	$\Delta\Delta G_{FEP9}$	$\Delta\Delta G_{calc}$ *(X→ Ala)*	$\Delta\Delta G_{calc}$ *(WT→ X)*	$\Delta\Delta G_{exp}$ *(Chen et al)*	$\Delta\Delta G_{exp}$ *(Cai et al)*
Tyr10$^{1.35}$	Tyr	6.3±0.9	7.1±0.3	-0.6±0.6	-0.2±0.1	0.4±0.3	0.1±0.2	0.1±0.6	-0.9±0.3	0.3±0.2	0.0±0.0		-0.9±0.9		ND	2.6
	Phe	-6.8±0.6	-7.0±0.7	0.1±0.1	0.0±0.1	-0.2±0.2	0.1±0.2	1.2±0.4	-1.0±0.4	0.2±0.2	-0.1±0.0		0.2±0.9	-1.1±1.3	1.2	ND
Ala64$^{2.61}$	Ile	-10.9±0.5	-12.7±0.5	-0.3±0.1	0.0±0.1	-0.1±0.2	0.2±0.3	1.8±0.4	0.1±0.0				1.8±0.7	-1.8±0.7	ND	2.8
Ser68$^{2.65}$	Ser	-7.2±0.6	-7.2±0.5	0.2±1.0	-0.3±0.0	0.2±0.1	0.0±0.0						0.0±0.8		0.4	0.2
Val85$^{3.32}$	Val	-5.5±0.3	-7.7±0.3	-0.2±0.0	-0.1±0.1	0.5±0.3	1.9±0.2	0.1±0.0					2.2±0.4		ND	1.6
Thr89$^{3.36}$	Thr	1.5±0.6	-1.7±0.4	2.3±0.6	-0.1±0.0	-0.2±0.1	1.3±0.3	-0.1±0.0					3.3±0.8		ND	2.2
Ile93$^{3.40}$	Ile	-1.9±0.1	-4.8±0.4	0.0±0.0	0.0±0.0	0.1±0.1	0.4±0.2	2.3±0.3	0.1±0.0				2.9±0.4		ND	2.8
Phe173^{ECL2}	Phe	-4.3±0.5	-3.9±0.3	-0.2±0.1	-0.1±0.1	-0.5±0.1	-0.1±0.3	-1.3±0.4	0.8±0.3	0.9±0.2	0.1±0.0		-0.4±0.6		ND	2.5
Met179$^{5.35}$	Met	-0.4±0.4	-0.6±0.4	0.0±0.2	0.0±0.0	0.3±0.2	-0.3±0.3	0.2±0.3	0.0±0.1	0.0±0.0			0.2±0.5		ND	0.7
Met182$^{5.38}$	Met	2.0±0.7	2.4±0.5	-0.7±0.4	-0.2±0.1	-0.2±0.1	-1.3±0.2	1.3±0.6	0.7±0.4	0.0±0.0			-0.4±0.8		ND	2.4
Asn186$^{5.42}$	Asn	36.9±0.4	37.6±0.4	-1.5±0.5	-0.4±0.1	0.0±0.2	1.1±0.2	0.0±0.0					-0.7±0.5		ND	0.0
Val191$^{5.47}$	Val	-3.5±0.2	-5.5±0.3	0.1±0.0	-0.2±0.1	0.3±0.2	1.7±0.4	0.1±0.0					2.0±0.4		ND	2.4
Trp247$^{6.48}$	Trp	13.6±0.4	8.1±0.9	-0.1±0.1	-0.3±0.1	0.6±0.2	-0.5±0.2	1.2±0.5	2.1±0.4	1.7±0.4	1.0±0.3	-0.2±0.1	5.5±1.0		ND	1.8
Val250$^{6.51}$	Val	-7.7±0.4	-7.4±0.4	-0.5±0.1	-0.4±0.1	0.0±0.4	0.4±0.3	0.2±0.1					-0.3±0.6		0.9	0.8
	Leu	-6.2±0.3	-6.2±0.4	-0.3±0.1	-0.6±0.2	0.8±0.3	0.4±0.2	0.2±0.0	0.0±0.0				0.0±0.5	-0.3±0.8	1.5	2.8
His251$^{6.52}$	His	19.2±0.8	17.8±0.8	1.5±0.6	0.0±0.0	-0.3±0.1	-0.9±0.3	0.6±0.4	0.5±0.4	0.0±0.1			1.4±1.1		ND	2.5
Asn254$^{6.55}$	Asn	36.9±0.5	35.4±0.4	1.0±0.3	-0.6±0.1	0.2±0.2	0.8±0.2	0.0±0.0					1.4±0.6		1.4	2.6
Met272$^{7.35}$	Met	-0.3±0.3	-0.9±0.3	-0.4±0.1	0.1±0.0	0.7±0.1	0.6±0.1	-0.6±0.3	0.2±0.1	0.0±0.0			0.5±0.5		ND	1.0
Asn273$^{7.36}$	Asn	32.6±0.2	32.9±0.1	-0.1±0.2	-0.4±0.0	-0.2±0.1	0.3±0.1	0.0±0.0					-0.3±0.3		1.1	ND
	Tyr	4.9±0.6	3.1±0.6	1.2±0.4	-0.3±0.1	-0.4±0.2	-0.2±0.2	0.1±0.2	0.7±0.4	0.6±0.2	0.0±0.0		1.8±0.8	-2.1±0.9	1.1	ND
Ile276$^{7.39}$	Ile	-13.4±0.7	-11.2±0.4	0.2±0.0	-0.4±0.1	-0.9±0.2	-1.3±0.4	0.2±0.6	-0.1±0.0				-2.2±0.8		ND	2.5
Ser279$^{7.42}$	Ser	-5.7±0.7	-4.6±0.9	-1.8±1.3	-0.2±0.0	0.8±0.1	0.1±0.0						-1.2±1.1		ND	1.6
His280$^{7.43}$	Hip	136.0±0.9	133.2±0.7	1.8±0.0	0.0±0.7	0.1±3.0	0.3±1.1	0.2±1.1	0.2±0.1	0.1±0.0			2.8±1.1		ND	2.7

[a] *Experimental relative binding free energies ($\Delta\Delta G_{exp}$) were calculated from EC_{50} values as* $\Delta\Delta G_{exp} = RTln(\frac{EC_{50}^{mut}}{EC_{50}^{wt}})$. *Calculated relative binding free energies ($\Delta\Delta G_{calc}$) are obtained from series of small, convergent FEP calculations* $((\Delta G_{holo}^{wt} - \Delta G_{apo}^{wt}) - (\Delta G_{holo}^{mut} - \Delta G_{apo}^{mut}))$

[b] *ND: not determined*
